# Epigenetic Remodeling of Meiotic Crossover Frequency in *Arabidopsis thaliana* DNA Methyltransferase Mutants

**DOI:** 10.1371/journal.pgen.1002844

**Published:** 2012-08-02

**Authors:** Nataliya E. Yelina, Kyuha Choi, Liudmila Chelysheva, Malcolm Macaulay, Bastiaan de Snoo, Erik Wijnker, Nigel Miller, Jan Drouaud, Mathilde Grelon, Gregory P. Copenhaver, Christine Mezard, Krystyna A. Kelly, Ian R. Henderson

**Affiliations:** 1 Department of Plant Sciences, University of Cambridge, Cambridge, United Kingdom; 2 Institut Jean-Pierre Bourgin, INRA Centre de Versailles-Grignon, Versailles, France; 3 The James Hutton Institute, Invergowrie, Dundee, United Kingdom; 4 Rijk Zwaan Breeding, De Lier, The Netherlands; 5 Wageningen University, Wageningen, The Netherlands; 6 Department of Pathology, University of Cambridge, Cambridge, United Kingdom; 7 Department of Biology and The Carolina Center for Genome Sciences, The University of North Carolina at Chapel Hill, Chapel Hill, North Carolina, United States of America; 8 Lineberger Comprehensive Cancer Center, The University of North Carolina School of Medicine, Chapel Hill, North Carolina, United States of America; Stanford University School of Medicine, United States of America

## Abstract

Meiosis is a specialized eukaryotic cell division that generates haploid gametes required for sexual reproduction. During meiosis, homologous chromosomes pair and undergo reciprocal genetic exchange, termed crossover (CO). Meiotic CO frequency varies along the physical length of chromosomes and is determined by hierarchical mechanisms, including epigenetic organization, for example methylation of the DNA and histones. Here we investigate the role of DNA methylation in determining patterns of CO frequency along *Arabidopsis thaliana* chromosomes. In *A. thaliana* the pericentromeric regions are repetitive, densely DNA methylated, and suppressed for both RNA polymerase-II transcription and CO frequency. DNA hypomethylated *methyltransferase1* (*met1*) mutants show transcriptional reactivation of repetitive sequences in the pericentromeres, which we demonstrate is coupled to extensive remodeling of CO frequency. We observe elevated centromere-proximal COs in *met1*, coincident with pericentromeric decreases and distal increases. Importantly, total numbers of CO events are similar between wild type and *met1*, suggesting a role for interference and homeostasis in CO remodeling. To understand recombination distributions at a finer scale we generated CO frequency maps close to the telomere of chromosome 3 in wild type and demonstrate an elevated recombination topology in *met1*. Using a pollen-typing strategy we have identified an intergenic nucleosome-free CO hotspot *3a*, and we demonstrate that it undergoes increased recombination activity in *met1*. We hypothesize that modulation of *3a* activity is caused by CO remodeling driven by elevated centromeric COs. These data demonstrate how regional epigenetic organization can pattern recombination frequency along eukaryotic chromosomes.

## Author Summary

The majority of eukaryotes reproduce via a specialized cell division called meiosis, which generates gametes with half the number of chromosomes. During meiosis, homologous chromosomes pair and undergo a process of reciprocal exchange, called crossing-over (CO), which generates new combinations of genetic variation. The relative chance of a CO occurring is variable along the chromosome, for example COs are suppressed in the centromeric regions that attach to the spindle during chromosome segregation. These patterns correlate with domains of epigenetic organization along chromosomes, including methylation of the DNA and histones. DNA methylation occurs most densely in the centromeric regions of *Arabidopsis thaliana* chromosomes, where it is required for transcriptional suppression of repeated sequences. We demonstrate that mutants that lose DNA methylation (*met1*) show epigenetic remodeling of crossover frequencies, with increases in the centromeric regions and compensatory changes in the chromosome arms, though the total number of crossovers remains the same. As crossover numbers and distributions are subject to homeostatic mechanisms, we propose that these drive crossover remodeling in *met1* in response to epigenetic change in the centromeric regions. Together these data demonstrate how domains of epigenetic organization are important for shaping patterns of crossover frequency along eukaryotic chromosomes.

## Introduction

During meiosis homologous chromosomes pair and undergo reciprocal exchange, to produce crossovers (COs). COs are initiated by SPO11-catalyzed DNA double strand breaks (DSBs), which are resected to generate single-stranded 3′ tails on either side of the break (ssDNA) [1]. The ssDNA can invade a non-sister chromatid to form an intermediate D-loop structure, which may proceed to form a double Holliday junction that can be resolved into a CO [1], [2], [3]. The D-loop can also participate in an alternative pathway to form non-crossovers (NCOs), which in *Saccharomyces cerevisiae* involves synthesis dependent strand annealing [1], [2], [3]. Concurrently with DSB generation a chromosome axis forms and physically connects the homologues with loops of chromatin projecting laterally [4], [5], [6]. DSBs arise on chromatin loops tethered to the axis, and changes to axis structure can dramatically alter recombination patterns [4], [6], [7]. A greater number of DSBs are generated than mature into COs, with the excess DSBs repaired as NCOs, some of which can be detected as gene conversions [8], [9]. COs occurring between homologous chromosomes can show distance-dependent interference causing them to be more widely spaced than expected by chance [9], [10]. For example, in *A. thaliana* 85–90% of COs form via the MSH4-dependent interfering pathway (type-I) and the remaining 10–15% form via the MUS81-dependent non-interfering pathway (type-II) [11], [12], [13], [14], [15]. Additional CO pathways must also exist in *A. thaliana* since residual COs or chiasmata have been observed in *msh4 mus81* double mutants [11], [14]. A process related to interference, called homeostasis, maintains CO frequency when DSBs are reduced [16]. Interference and homeostasis cause CO number per chromosome to be distributed closer to a mean than expected from the Poisson distribution [13], [17]. Tight control of CO frequency is thought to be important because balanced homologue segregation at meiosis-I is dependent, in most organisms, on each pair of homologues having at least one CO [18].

**Figure 1:**
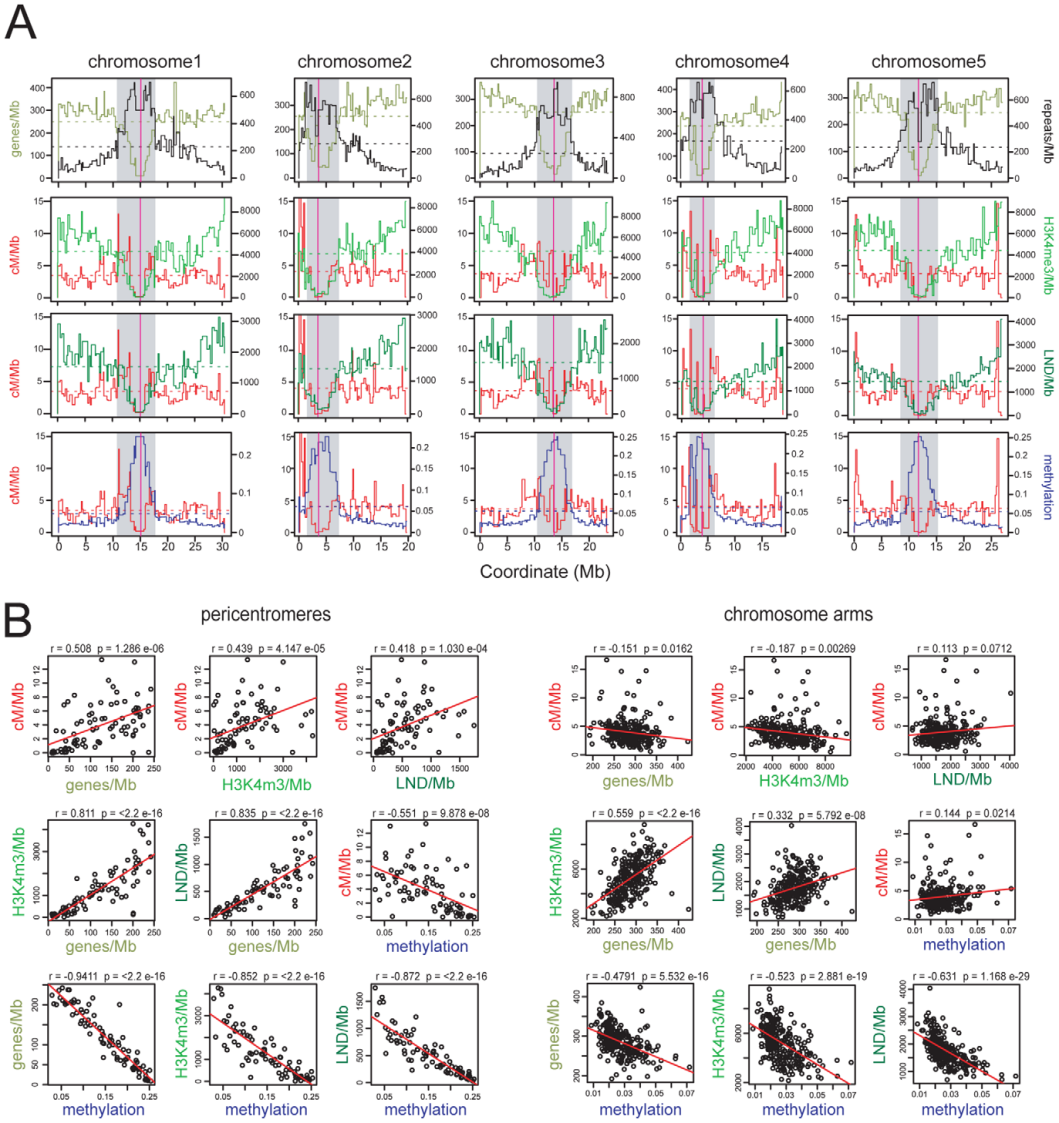
Epigenomic organisation and CO frequency in the *A. thaliana* genome. (A) Physical maps of *A. thaliana* chromosomes showing genes/Mb (olive green), repeats/Mb (black), cM/Mb (red), H3K3me3/Mb (light green), LND/Mb (dark green) and DNA methylation density (blue). Dotted horizontal lines indicate the means weighted by intermarker distance. Vertical magenta lines indicate the centromeres. Grey shaded areas indicate the pericentromeres. (B) Pairwise correlations between cM/Mb, genes/Mb, H3K4me3/Mb, LND/Mb and DNA methylation in either chromosome arms or pericentromeres. Pearson's correlation coefficients (r) and associated p-values (p) are shown and regression lines are plotted in red. See also Tables S1 and S2.

CO frequency is variable along the length of *A. thaliana* chromosomes, for example the centromeres are CO suppressed, whereas gene-dense regions are active [19], [20], [21], [22]. *A. thaliana* chromosomes also display region-specific epigenetic modifications of DNA and histones that are associated with differential transcription [23], [24], [25], [26], [27], [28], [29], [30]. DNA cytosine methylation is an epigenetic modification that can be heritably maintained through DNA replication and in *A. thaliana* occurs in two major epigenomic contexts. First, the majority of DNA methylation overlaps with RNA polymerase II (Pol II) repressed repetitive sequences including transposons and also with histone H3K9me2, H3K27me1, H4K20me1 (me = methylation) [23], [24], [25], [26], [29], [30], [31], [32]. Repeats are DNA methylated in all sequence contexts (CG, CHG and CHH) and show a marked increase in density towards the centromeres [23], [24], [25], [29], [30] (Figure 1). In the second context, the open reading frames of Pol II transcribed genes contain CG methylation, coincident with overlapping peaks of histone H3K4me, me2, me3, H3K36me3, H3K56ac and H2Bub (ac = acetylation, ub = ubiquitination) [26], [27], [29], [30], [33], [34]. Epigenetic information is known to influence patterns of meiotic recombination. For example, in *S.cerevisiae* and mammals CO hotspots associate with ‘accessible’ chromatin modifications, including histone H3K4me3 [35], [36], [37], [38], [39], [40], [41], and DNA methylation can directly repress COs in *Ascobolus immersus* [42]. Here we investigate the role of DNA methylation in organizing patterns of meiotic recombination frequency in the *A. thaliana* genome.

Maintenance of CG DNA methylation in *A. thaliana* requires the cytosine methyltransferase METHYLTRANSFERASE1 (MET1) [43], [44], [45], [46]. *A. thaliana met1* mutants show dramatic loss of DNA methylation and associated histone modifications, leading to increased Pol II transcription of repetitive sequences [25], [29], [30], [44], [45], [46], [47], [48]. Gene body DNA methylation is also lost in *met1*, though expression of these genes is maintained [25], [29], [30]. Self-fertilization and inbreeding of *met1* mutants leads to stochastic generation of epialleles and transposon mobilization [44], [45], [46], [49], [50], [51], [52], [53], [54]. Epigenetic divergence is observed in within *met1^+/−^* segregating populations, even without *met1* homozygosity, as plant haploid gametophytes undergo post-meiotic DNA replication and in *met1* gametophytes this causes cytosine demethylation [44], [46], [52]. Here we demonstrate extensive remodeling of CO distributions in *met1* mutants, with elevated centromere-proximal COs coupled to pericentromeric decreases and distal increases. Importantly total CO numbers are similar between wild type and *met1*, suggesting that interference and homeostasis may act to drive regional changes. We generate a fine-scale map of euchromatic recombination frequency close to the telomere of chromosome 3 and identify a novel, intergenic CO hotspot *3a*. We observe an elevated recombination topology across this region in *met1* and higher *3a* CO frequency, consistent with remodeling modulating hotspot activity. Together this work reveals the importance of domains of epigenetic organization in determining chromosomal patterns of meiotic CO frequency.

## Results

### Epigenetic organization and CO frequency in the *A. thaliana* genome

Because CO frequency is decreased close to the *A. thaliana* centromeres we investigated its relationship with DNA methylation in these regions [19], [20], [21], [22]. To obtain a genome-wide map of CO frequency we analyzed published genotype data for 17 F_2_ populations, providing a total dataset of 55,497 COs [22], [55]. Genetic maps for individual populations were created using R/qtl and merged using MergeMap, which yielded map lengths comparable to those previously published (Table S1) [21], [22], [56], [57], [58], [59]. We then calculated recombination frequency (cM/Mb), gene, H3K4me3, LND (low nucleosome density), repeat and DNA methylation densities within marker intervals of the merged map. Meiosis-specific epigenomic maps are not currently available in *A. thaliana*, so bisulfite sequencing data (DNA methylation) and ChIP-chip data (H3K4me3 and LND) generated from somatic tissues were used [23], [27], [28] (Table S2).

We defined pericentromeres as the intervals flanking the genetically defined centromeres that showed gene densities lower than the chromosome average, and defined the remaining regions as chromosome arms (Figure 1 and Table S2) [19]. The pericentromeres contain fewer genes, higher repetitive DNA content and denser DNA methylation compared to the chromosome arms (averages for chromosome arms vs pericentromeres are 286.9 vs 123.6 genes/Mb, 153.4 vs 556.4 repeats/Mb, 0.027 vs 0.147 for methylation). Gene density is positively correlated with H3K4me3 and LND density in all regions, consistent with the known function of these chromatin features in promoting gene expression (Figure 1B) [27], [28]. Gene, H3K4me3 and LND density are negatively correlated with DNA methylation, most strongly in the pericentromeres, consistent with dense DNA methylation associating with Pol II silenced repeats (Figure 1B) [23], [24], [25], [29], [30]. Mean CO frequencies within the chromosome arms (3.95 cM/Mb) and pericentromeres (3.83 cM/Mb) were similar, though within the pericentromeres CO frequency was strongly elevated towards the region boundaries (Figure 1A), and showed positive correlations with genes/Mb (r = 0.508, p = 1.29×10^−06^), H3K4me3/Mb (r = 0.439, p = 4.15×10^−05^), LND/Mb (r = 0.418, p = 1.03×10^−04^) and a negative correlation with DNA methylation (r = −0.551, p = 9.88×10^−08^) (Figure 1B). In contrast, cM/Mb in the chromosome arms was weakly correlated with genes/Mb, H3K4me3/Mb, LND/Mb and methylation (Figure 1B). This indicates that pericentromeres represent chromosomal domains with distinct patterns of epigenetic information and CO frequency control relative to the chromosome arms. Given the negative correlation between DNA methylation and CO frequency within the pericentromeres we decided to test CO patterns in hypomethylated *met1–3* mutants [23], [25], [29], [30].

### Elevated centromeric CO frequency in *met1–3*

**Figure 2:**
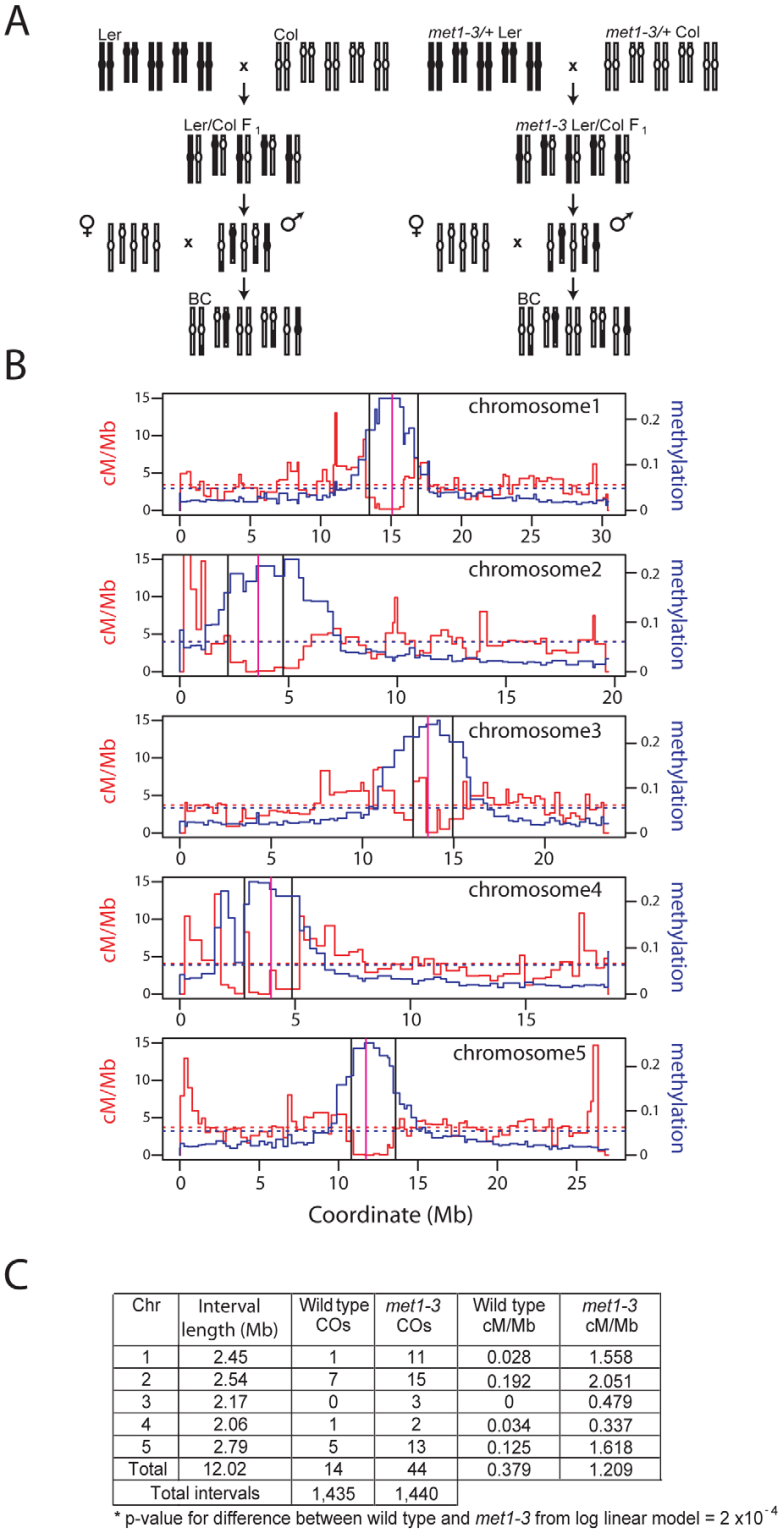
Elevated centromeric crossovers in *met1–3.* (A) Schematic diagram illustrating generation of wild type and *met1–3^−/−^* recombinant male backcross populations from Col and Ler homozygous parents. (B) Chromosome physical maps with overlaid cM/Mb (red) and DNA methylation (blue) plots; black vertical lines indicate the position of polymorphic Col/Ler markers tested for segregation frequency. Vertical magenta lines indicate centromeres. (C) Segregation data and centromeric CO measurements in wild type and *met1–3^−/−^* male backcross populations.

To measure COs in proximity of the centromeres in *met1* we analyzed the segregation of polymorphic markers (Figure 2A). We backcrossed the null *met1–3* allele from the Columbia (Col) accession into Landsberg *erecta* (Ler) for 8 generations, maintaining *met1–3* as a heterozygote to limit epigenetic divergence. *met1–3^+/−^* ^Ler^ and *met1–3^+/−^* ^Col^ heterozygotes were crossed to generate F_1_ individuals homozygous for *met1–3* and heterozygous for Col/Ler polymorphisms. To generate recombinant populations these F_1_ individuals were backcrossed as males to Col, as were wild type Col/Ler heterozygotes (Figure 2A). We designed insertion-deletion Col/Ler PCR markers to centromere proximal positions that show CO suppression and dense DNA methylation (Figure 2B and 2C). We observed significantly elevated centromere-proximal CO frequency in the mutant *met1–3^−/−^* population relative to wild type (1.21 cM/Mb vs 0.38 cM/Mb, p_mod_ = 2.0×10^−4^) (Figure 2C). As expected wild type recombination rates within these densely DNA methylated regions were lower than the chromosome averages (Figure 2C and Table S1). These data demonstrate elevated centromere-proximal COs in *met1–3^−/−^*, correlating with extensive DNA demethylation and increased Pol II transcription previously observed in these regions [23], [25], [29], [30].

### Stochastic decrease of pericentromeric CO frequency in *met1–3*

**Figure 3:**
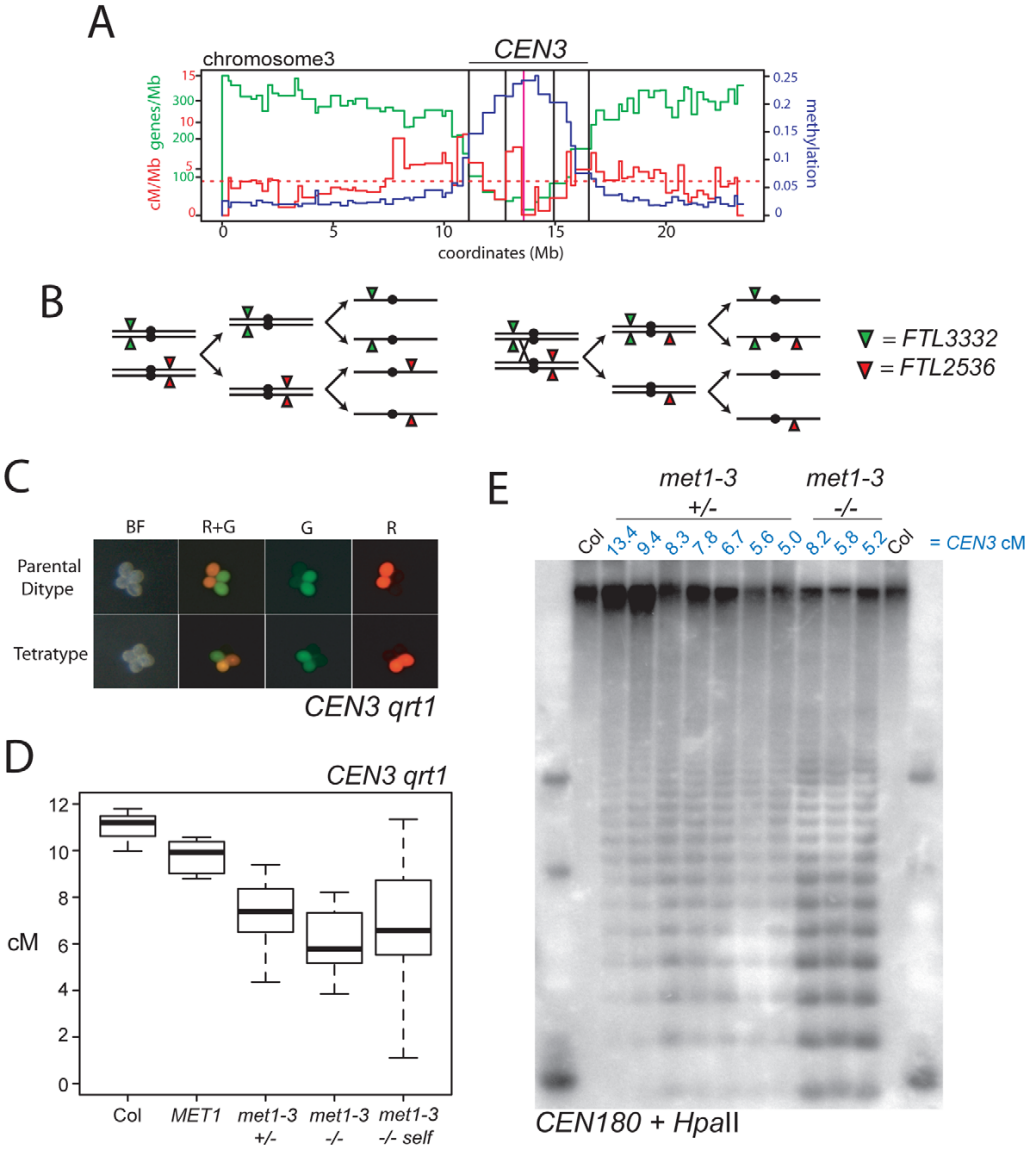
Decreased pericentromeric crossovers in *met1–3*. (A) Physical map of chromosome 3 with overlaid genes/Mb (green), cM/Mb (red) and DNA methylation (blue) plots. The dotted, horizontal red line indicates the cM/Mb weighted mean. Outer vertical black lines indicate the position of FTL transgene insertions that define *CEN3*. Inner vertical black lines indicate the position of centromeric markers analyzed in Figure 2. The vertical magenta line indicates the centromere. (B) Chromosomes heterozygous for *trans-*linked *FTL332* (eYFP) and *FTL2536* (DsRed) transgenes, which flank the centromere (black circle) segregating through meiosis-I and –II in the absence (left) or presence (right) of a CO within *CEN3*. (C) Fluorescence micrographs of *qrt1–2* pollen showing patterns of inheritance associated with (tetratype) or without (parental ditype) a CO within *CEN3*. BF shows bight field illumination and R and G indicate red and green UV fluorescence. (D) *CEN3* genetic map lengths for naïve wild type (Col), *MET1*, *met1–3^+/−^*, *met1–3^−/−^* segregants and self-fertilized *met1–3^−/−^* measured by *qrt1–2^−/−^* tetrad counting. (E) Southern blotting and hybridization analysis of *CEN180* following digestion of genomic DNA using DNA methylation sensitive *HpaII*. DNA was prepared from *CEN3 qrt1–2^−/−^* individuals whose measured genetic distance in cM is indicated above the blot in blue in addition to their *met1–3* genotype. See also Table S3.

We sought to test CO frequency in wild type and *met1–3^−/−^* across a wider pericentromeric interval. The FTL system uses segregation of heterozygous transgenes expressing distinct colors of fluorescent proteins in pollen to measure COs between insertion sites [60] (Figure 3). *FTL* segregation in the *quartet1–2* (*qrt1–2*) mutant background, where sister pollen grains remain physically attached, allows tetrad analysis for male meioses [60] (Figure 3B and 3C). We used FTL lines located on chromosome 3 defining a 5.405 Mb interval that we call *CEN3*, which spans the centromere and includes the region previously measured in the backcross populations, in addition to flanking pericentromeric DNA (Figure 3A). *CEN3* is repeat and methylation dense (650.8 repeats/Mb, 0.183 methylation) and gene-poor (75.1 genes/Mb) compared to the chromosome 3 averages (240.7 genes/Mb, 273.7 repeats/Mb, 0.056 methylation). In Col that has never been crossed to *met1–3* (naïve wild type) *CEN3* has a genetic distance of 11.04 cM, corresponding to 2.05 cM/Mb, compared to the 4.76 cM/Mb chromosome 3 male average (Figure 3D and Tables S1 and S3) [21]. Although *CEN3* is relatively suppressed for COs, this interval shows increasing CO frequency towards its boundaries, correlating with higher gene densities and lower DNA methylation (Figure 3A).

We self-fertilized *CEN3/−− met1–3^+/−^ qrt1–2^−/−^* plants to generate populations segregating for *met1–3* and measured *CEN3* COs in *MET1*, *met1–3^+/−^* and *met1–3^−/−^* individuals. We observed significant decreases in *CEN3* genetic distance in all groups relative to naïve wild type, with mean distances of *MET1* 9.76 cM (p_t_ = 0.01), *met1–3^+/−^* 7.32 cM (p_t_ = 4.31×10^−5^) and *met1–3^−/−^* 6.68 cM (p_t_ = 0.002) (Figure 3D and Table S3). After self-fertilization *met1–3^−/−^* maintained a significantly decreased *CEN3* mean genetic distance of 6.37 cM (p_t_ = 0.001) (Figure 3D and Table S3). The *met1–3^+/−^* and *met1–3^−/−^* self-fertilized segregant groups also exhibited significantly greater variability in CO frequency compared to naïve wild type (F-test: *met1–3^+/−^* p = 0.0152 and *met1–3^−/−^* p = 4.32e-3) (Figure 3D and Table S3). Increased variance is consistent with stochastic epigenetic divergence observed in segregating *met1* and *ddm1* populations [44], [46], [50], [52], [61], [62], [63]. These data are consistent with increased centromere-proximal COs in *met1–3^−/−^* (*met1–3^−/−^* 1.21 cM/Mb vs wild type 0.38 cM/Mb) decreasing CO frequency in pericentromeric regions (*met1–3^−/−^* 1.24 cM/Mb vs wild type 2.05 cM/Mb), potentially via CO interference.

We investigated whether centromeric DNA methylation correlates with *CEN3* genetic distance in this population. To analyze centromeric DNA methylation we used methyl-sensitive restriction digestion of genomic DNA with *Hpa*II followed by Southern blotting and hybridization with the *A. thaliana* 180-bp satellite repeat *CEN180* (Figure 3E) [48]. The 180-bp satellite repeats occur in tandem arrays of megabase length within centromeres and are densely DNA methylated in wild type [19], [48], [64]. In *met1–3^−/−^* mutants the satellite repeats lose methylation and are digested by *Hpa*II, whereas wild type Col DNA is undigested (Figure 3E). We analyzed leaf DNA from *met1–3^+/−^* and *met1–3^−/−^* individuals for which we had measured *CEN3* genetic distance. We observed that greater satellite demethylation was associated with decreased *CEN3* recombination, though two *met1–3^+/−^* individuals (5.6 cM and 5.0 cM) deviated from this trend (Figure 3E). This may be explained by chromosome 3 being demethylated to a greater extent than other chromosomes in these lines. These data demonstrate decreased pericentromeric CO frequency in *met1–3* mutants, coincident with DNA demethylation of the satellite repeats. This is consistent with CO interference from elevated centromere-proximal COs reducing events closer to the boundaries of *CEN3*.

### Total genetic map length is similar between wild type and *met1–3*

**Figure 4:**
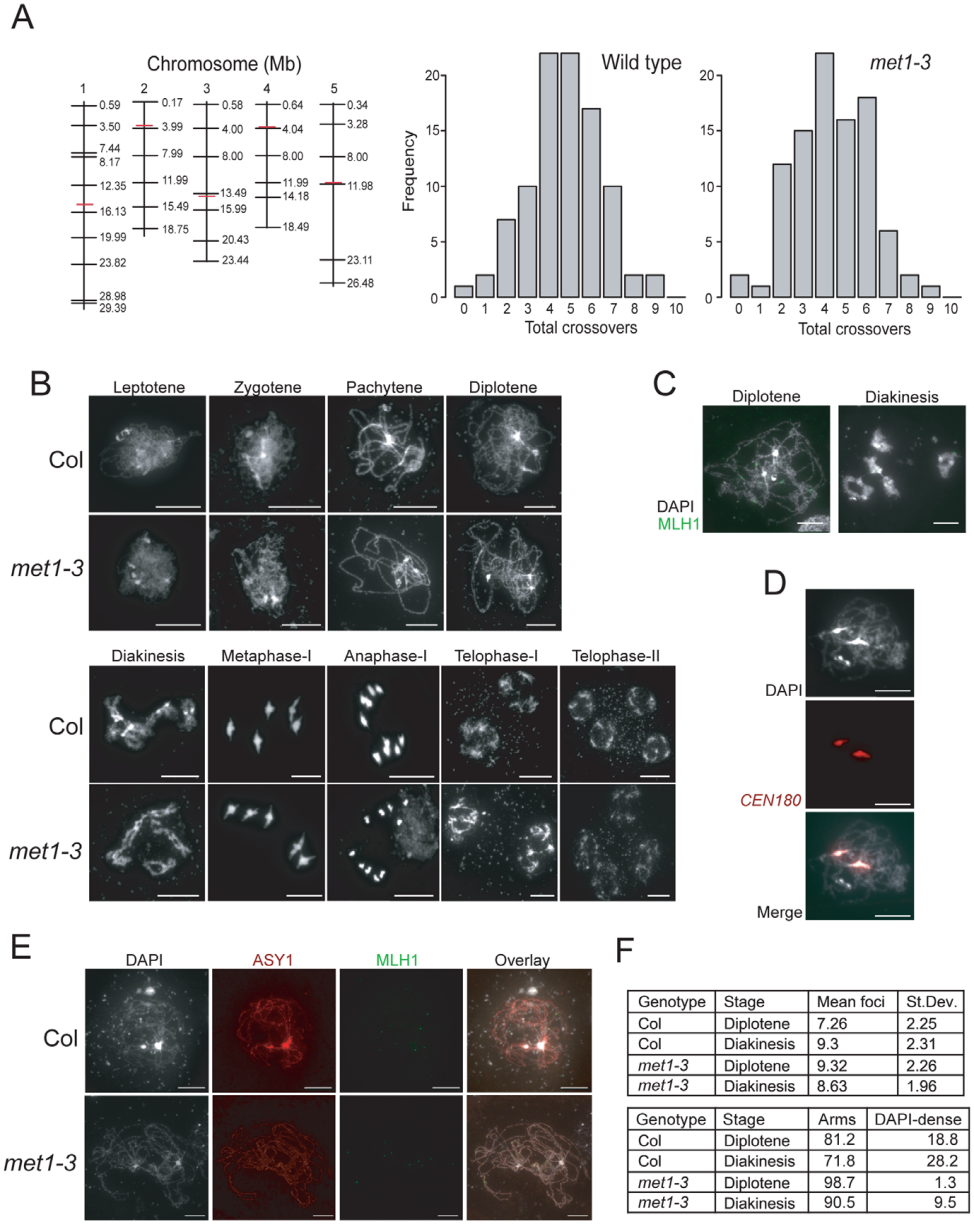
Total crossover numbers are similar between wild type and *met1–3.* (A) Physical maps of chromosomes (vertical black lines) with KASPar marker (horizontal black lines) and centromere (horizontal red lines) positions indicated. Histograms showing the frequency of total CO numbers identified in male backcross individuals from either Col/Ler F_1_ (wild type) or *met1–3^−/−^* Col/Ler F_1_ (*met1–3*) parents. (B) Micrographs of DAPI-stained anther meiocytes showing the labeled stage of meiosis in Col and *met1–3^−/−^*. (C) Micrographs of diplotene and diakineses stage male meiocytes stained with DAPI (white) and immunostained for MLH1 (green). (D) Micrographs showing co-localisation of dense-DAPI staining and *in situ* hybridization with the *CEN180* satellite repeat (red). (E) Micrographs of male meiocytes stained with DAPI (white) and immunostained with MLH1 (green) and the axis component ASY1 (red). (F) The upper table lists mean MLH1 foci numbers in wild type and *met1–3^−/−^* at diplotene or diakinesis with standard deviation (+/−). The lower table lists the relative proportions (%) of MLH1 foci localizing to chromosome arm regions (arms) vs densely-DAPI staining regions (DAPI-dense). All scale bars represent 10 µM. See also Table S4 and S5.

Total CO numbers in *A. thaliana* do not follow the Poisson distribution, indicating homeostatic control [12], [13], [21], [57]. We therefore tested whether total genetic map length in *met1–3^−/−^* was different from wild type, given our observations that regional frequencies close to the centromeres were altered. To measure map length we genotyped 95 male backcross individuals, generated from wild type or *met1–3^−/−^* Col/Ler heterozygotes, for 35 Col/Ler SNPs spaced across the 5 chromosomes using KASPar technology (Figure 4A and Table S4) [65], [66]. Total CO numbers were not significantly different between wild type and *met1–3^−/−^* populations (p_mod_ = 0.13) (Figure 4A and Table S4). Therefore, despite regional alterations in CO frequency, total genetic map length is similar between *met1–3^−/−^* and wild type.

To investigate meiotic progression in more detail we performed DAPI staining of anther meiocytes in wild type and *met1–3^−/−^*. The major cytological stages of meiosis in *met1–3^−/−^* lacked dramatic alterations to chromosome morphology or segregation (Figure 4B). At leptotene replicated chromosomes were present as thin threads, which condensed during zygotene, and became fully synapsed by pachytene (Figure 4B). At pachytene the centromeres, pericentromeres and nucleolar organizing regions (NORs) cluster into densely DAPI-staining regions, which remain evident in *met1–3^−/−^* [67] (Figure 4B). During diplotene desynapsis occurs and homologues begin to separate, which further condense during diakinesis, when chiasma connecting the homologues are evident (Figure 4B). At metaphase-I bivalents are maximally condensed with homologous centromeres segregating to opposite cell poles. Segregation forms cell dyads, each containing 5 homologues, which partially decondense at telophase-I (Figure 4B). The second meiotic division separates chromatids, which decondense to form haploid tetrads at telophase-II (Figure 4B). This analysis demonstrates that overall meiotic chromosome morphology and segregation are similar between wild type and *met1–3^−/−^*.

As an independent measure for CO numbers we immunostained wild type and *met1–3^−/−^* meiocytes for MLH1, which is a homolog of bacterial MutL DNA repair proteins and localizes to foci corresponding to type-I (interference sensitive) COs (Figure 4C and Table S5) [17]. MLH1 foci are first detected at pachytene and increase to maximal numbers during diplotene and diakinesis (Figure 4C) [17]. MLH1 foci are closely associated with the chromosomes, visualized by either DAPI-staining or immunostaining for the axis component ASY1 (Figure 4C and 4E) [68]. We counted MLH1 foci from diplotene and diakinesis stage meiocytes in wild type and *met1–3^−/−^*. At diplotene there were significantly more MLH1 foci in *met1–3^−/−^* relative to wild type (wild type mean = 7.26, *met1–3^−/−^* mean = 9.32, p_mod_ = 9.1 e-4) (Figure 4F and Table S5), though by diakinesis MLH1 numbers were not significantly different (wild type mean = 9.32, *met1–3^−/−^* mean = 8.63, p_mod_ = 0.39) (Figure 4F and Table S5). These data are consistent with total MLH1 foci numbers being similar between *met1–3^−/−^* and wild type, though maximal numbers may be reached slightly earlier in *met1–3^−/−^*.

Previous work demonstrated that MLH1 foci show differential localization on chromosome arms (77%) versus DAPI-dense regions (23%) at diakinesis [17]. We confirmed that these DAPI-dense regions contain the centromeres using fluorescent *in situ* hybridization for the *CEN180* satellite repeats (Figure 4D). We scored MLH1 foci distributions in wild type Col and observed similar results at diplotene (81.2% arms vs 18.8% DAPI-dense regions) and diakinesis (71.8% arms vs 28.2% DAPI-dense regions) (Figure 4E). In contrast, there were significantly fewer MLH1 foci in the DAPI-dense regions in *met1–3^−/−^* at both diplotene (98.7% arms vs. 1.3% DAPI-regions, chi-square p = 2.2 e-16) and diakinesis (90.5% arms vs. 9.5% DAPI-regions, chi-square p = 6.0 e-4) (Figure 4E). Together we interpret these data as indicating that although overall MLH1 foci numbers are similar between wild type and *met1–3^−/−^*, there are significantly fewer foci in the DAPI-dense regions in *met1–3^−/−^*. As DAPI-dense regions contain the pericentromeres, we interpret reduced MLH1 foci in these regions as reflecting the reduced pericentromeric genetic distance we observe over *CEN3* (Figure 3).

As we propose that CO interference mediates CO frequency remodeling in *met1–3^−/−^* we investigating whether interference occurred to a similar degree between wild type and *met1–3^−/−^*. To compare CO interference strength we calculated the average distance between pairs of COs identified from marker segregation occurring on the same chromosome (Double COs, DCOs) in the male backcross population described above (Table S4). The inter-CO distances and therefore the strength of CO interference were not significantly different between wild type and *met1–3^−/−^* (p_w_ = 0.67) (Table S4). As an additional measure of CO control we tested our MLH1 foci data for deviation from the Poisson distribution, which may indicate the action of CO interference [13], [69]. Using a goodness-of-fit test we observed significant deviations in all cases, with more MLH1 counts close to the mean than expected from the Poisson distribution (Table S5). This is consistent with interference acting in both wild type and *met1–3^−/−^*, supporting the idea that CO interference could contribute to the observed CO frequency remodeling in *met1–3^−/−^*. Together these data demonstrate that despite alteration of regional CO frequencies, total CO numbers and interference strength are similar between wild type and *met1–3^−/−^*. This is consistent with CO interference mediating inhibition of pericentromeric COs in *met1–3^−/−^*, due to elevated centromeric COs.

### Elevated euchromatic CO frequency in *met1–3*

**Figure 5:**
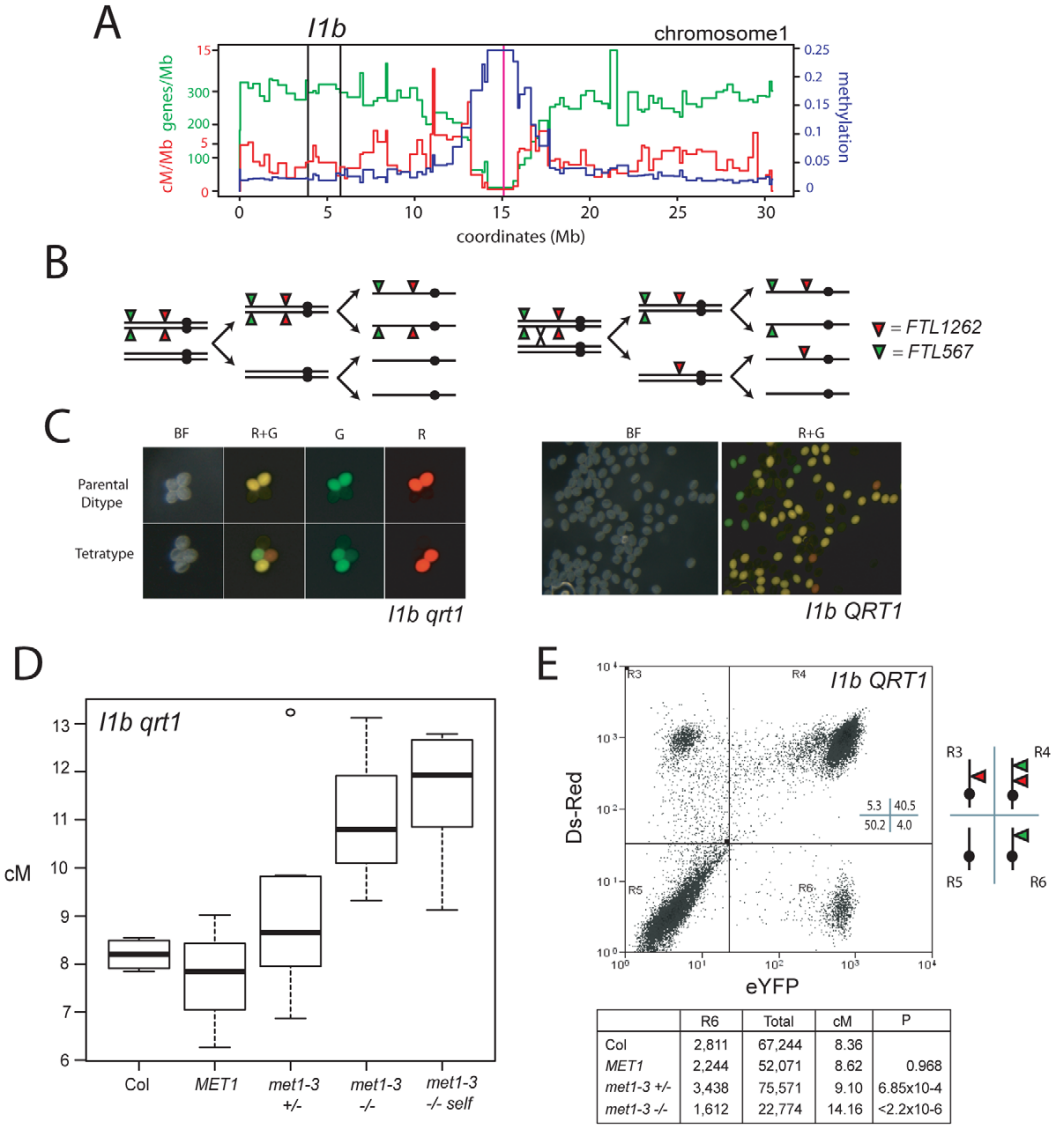
Elevated euchromatic crossovers in *met1–3*. (A) Physical map of chromosome 1 with overlaid gene/Mb (green), DNA methylation (blue) and cM/Mb (red) plots. Black vertical lines indicate the *I1b* transgene insertions and the magenta vertical line indicates the centromere. (B) Schematic diagram showing homologous chromosomes (black lines) heterozygous for *cis-*linked *FTL567* (eYFP) and *FTL1262* (RFP) transgenes segregating through meiosis in the absence or presence of a CO. (C) Fluorescence micrographs showing *qrt1–2^−/−^* or *QRT1* pollen from *I1b cis*-heterozygotes. (D) *I1b* genetic map length for naïve wild type (Col), *MET1*, *met1–3^+/−^* and *met1–3^−/−^* segregants and self-fertilized *met1–3^−/−^* (*met1-self*) measured by *qrt1–2^−/−^* tetrad counting. (E) *I1b* genetic map length for naïve wild type (Col) and *MET1*, *met1–3^+/−^* and *met1–3^−/−^* segregants measured by flow cytometry of individual pollen grains. A representative flow cytometry histogram from an *I1b cis*-heterozygote together with gate quadrant R6 counts, adjusted total pollen counts and cM. See also Table S6.

Given that we observed remodeling of centromere-associated CO frequencies in *met1–3^−/−^*, we next measured genetic distance in the euchromatic chromosome arms. The 1.85 Mb FTL *I1b* interval is relatively gene dense (310.8 genes/Mb) and repeat and methylation poor (84.3 repeats/Mb, 0.022 methylation) compared to the chromosome 1 averages (246.8 genes/Mb, 233.5 repeats/Mb, 0.048 methylation) (Figure 5A). *I1b* in naïve wild type measures 8.16 cM, and has a recombination rate in male meiosis of 4.41 cM/Mb, close to the chromosome 1 average (4.88 cM/Mb) (Figure 5D and Tables S1 and S6) [21]. In a population segregating for *I1b* and *met1–3* we observed that *met1–3^−/−^* individuals showed significantly increased genetic distance of 11.00 cM (5.95 cM/Mb) compared to naïve wildtype, *MET1* and *met1–3^+/−^* (p_t_ = 0.001, 0.03 and 0.08 respectively) (Figure 5D and Table S6). Elevated CO frequencies were stable when *met1–3^−/−^* plants were self-fertilized and measured in the next generation (Figure 5D and Table S6). Mean *I1b* CO frequencies of *met1–3^+/−^* (9.07 cM) segregants were higher than naïve wild type, though not significantly (p_t_ = 0.27). The *met1–3^+/−^*, *met1–3^−/−^* and *met1–3^−/−^* self-fertilized groups also had significantly higher variance relative to naïve wild type, consistent with epigenetic divergence (F-test: *met1–3^+/−^* p = 0.011, *met1–3^−/−^* p = 0.0447 and *met1–3^−/−^* self-fertilized p = 0.0445) (Figure 5D and Table S6).

We confirmed these observations after backcrossing *I1bc qrt1–2^−/−^* to either Col or *met1–3^−/−^* to complement with *QRT1* and used flow cytometry to measure the fluorescence of individual pollen grains (Figure 5C, 5E and Figure S1). The *I1b FTL* transgenes are *cis-*linked, meaning pollen from *I1b/−−* heterozygotes expressing red-alone or yellow-alone represent single CO events (Figure 5E and Figure S1). The recombination rate is calculated by the ratio of yellow-alone pollen grains to an adjusted total (Text S1 and Figure S1). In naïve wild type this technique measured an *I1b* genetic distance (8.16 cM) close to that observed from *qrt1–2^−/−^* tetrad scoring (8.20 cM) (Figure 5D and 5E). Both *met1–3^+/−^* and *met1–3^−/−^* plants showed significantly increased genetic distances of 9.10 cM (p_t_ = 6.85×10^−4^) and 14.16 cM (p_t_<2.20×10^−16^) respectively, whereas *MET1* segregants were not significantly different from naïve wild type (Figure 5D and 5E). These results confirm that *met1–3* CO frequency is elevated within *I1b*.

**Figure 6:**
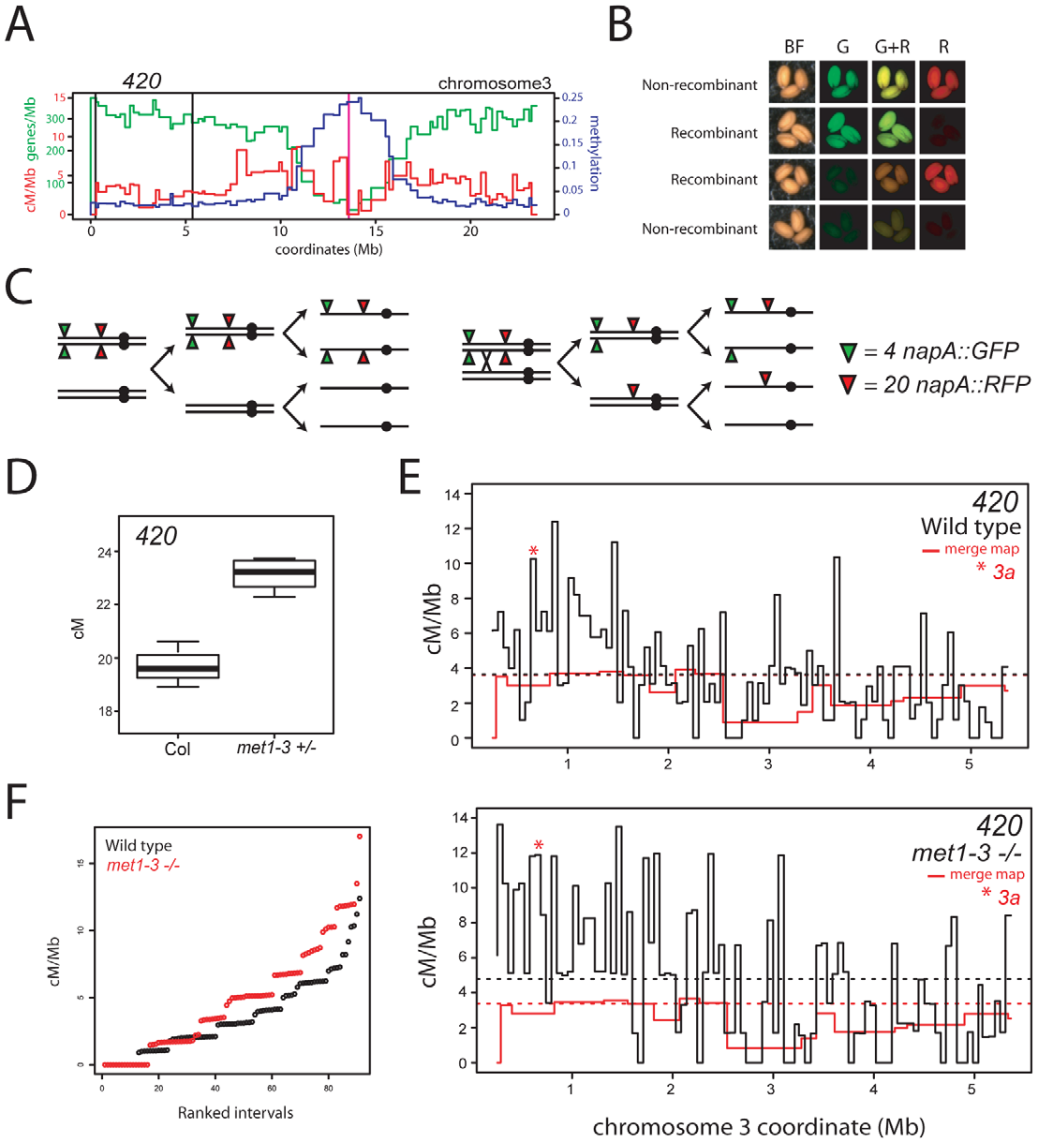
Elevated euchromatic recombination topology in wild type and *met1–3.* (A) Physical map of chromosome 3 with overlaid gene/Mb (green), DNA methylation (blue) and cM/Mb (red) plots. Black vertical lines indicate the positions of *napA* transgene insertions that define the *420* interval and the vertical magenta line indicates the centromere. (B) Fluorescence micrographs of seed expressing different combinations of *napA* transgenes. (C) Segregation diagram showing *cis-*heterozygous arrangement of *420 napA* lines. (D) *420* genetic distance measured in naïve wild type (Col) and *met1–3^+/−^* segregants. (E) Black lines indicate recombination frequency (cM/Mb) maps of the *420* interval in wild type or *met1–3^−/−^* with horizontal dotted lines indicating weighted means. Red lines represent merged map recombination frequency data for the *420* interval. The red star indicates the interval containing the *3a* CO hotspot. (F) Plots showing cumulative recombination value (cM/Mb) of ranked *420* mapping intervals in wild type (black) and *met1–3^−/−^* (red). See also Tables S7, S8 and S9.

To test the effect of *met1–3* on a second euchromatic interval we used a seed reporter system (Col3–4/20, hereafter referred to as *420*) [70] (Figure 6A, 6B and 6C). The *420* interval is defined by transgene insertions on chromosome 3 expressing GFP or RFP in seed from the *NapA* promoter [70] (Figure 6A). *420* spans 5.105 Mb and is relatively gene dense (311.9 genes/Mb) and repeat and methylation poor (71.5 transposons/Mb, 0.022 methylation) compared to the chromosome 3 averages (240.7 genes/Mb, 273.7 repeats/Mb, 0.056 methylation). In naïve, self-fertilised Col *420* has a mean genetic distance of 19.71 cM and recombination rate of 3.86 cM/Mb (chromosome 3 average 3.73 cM/Mb) (Figure 6D and Tables S1 and S7) [21]. We observed significant increases in mean *420* cM in *met1–3^+/−^* segregants to 23.32 cM (p_t_ = 0.004), relative to naïve wild type (Figure 6D and Table S7). These data confirm that CO frequency is elevated in the distal chromosome arms in *met1–3^+/−^* populations.

### Elevated euchromatic recombination topology in *met1–3*

To compare wild type and *met1–3* CO distributions at higher resolution we generated recombination frequency maps within the *420* interval. *420/−−* Col/Ler F_1_ hybrids, that were wild type or *met1–3^−/−^*, were backcrossed to Col as males and seed expressing red or green fluorescence alone were selected to identify recombinants within the *420* interval (Figure 6B and 6C). The *420* interval is strongly heterochiasmic with significantly higher male CO frequency (4.82 cM/Mb) than female (2.57 cM/Mb) (p_t_ = <2.20×10^−8^) (Table S8) [70], [71]. Male and female *420* genetic distances are reduced in Col/Ler heterozygotes compared to Col/Col homozygotes, potentially due to inhibition of recombination by polymorphisms (Table S8) [72]. CO frequency within *420* is significantly elevated by *met1–3^−/−^* in both Col/Col and Col/Ler backgrounds (Figure 6D, Tables S7 and S8), indicating that euchromatic remodeling is not dependent upon polymorphism levels.

We used an Illumina BeadArray to genotype 91 internal Col/Ler SNPs (average interval 56,067 bp) in 337 wild type and 268 *met1–3^−/−^ 420* recombinants (Table S9). Pronounced heterogeneity in cM/Mb was observed between intervals (range = 0–17.03 cM/Mb) with overall CO rate elevated in the *met1–3^−/−^* map relative to wild type (Figure 6E, 6F and Table S9). Recombination frequency topology was similar in both maps and showed significant correlation (r = 0.513, p = 1.95×10^−7^), and this correlation was stronger when comparisons were made over 255 Kb intervals (r = 0.789, p = 2.07×10^−8^). Elevated CO rates were observed towards the telomere in both populations (correlation between interval start coordinate and cM/Mb: wild type r = −0.496, p = 5.82×10^−7^; *met1–3^−/−^* r = −0.533, p = 5.21×10^−7^), consistent with higher telomeric CO rates observed in *A. thaliana* male meiosis relative to female (Figure 6A and Table S9) [20], [21], [57], [71], [73], [74], [75]. No significant correlations were detected between wild type cM/Mb and gene (r = −0.008, p = 0.94) or repeat (r = 0.005, p = 0.96) density at this scale. The similarity in overall recombination topology between wild type and *met1–3^−/−^* maps is consistent with remodeling acting to elevate existing CO patterns within *420*.

### Elevated CO hotspot *3a* activity in *met1–3*

**Figure 7:**
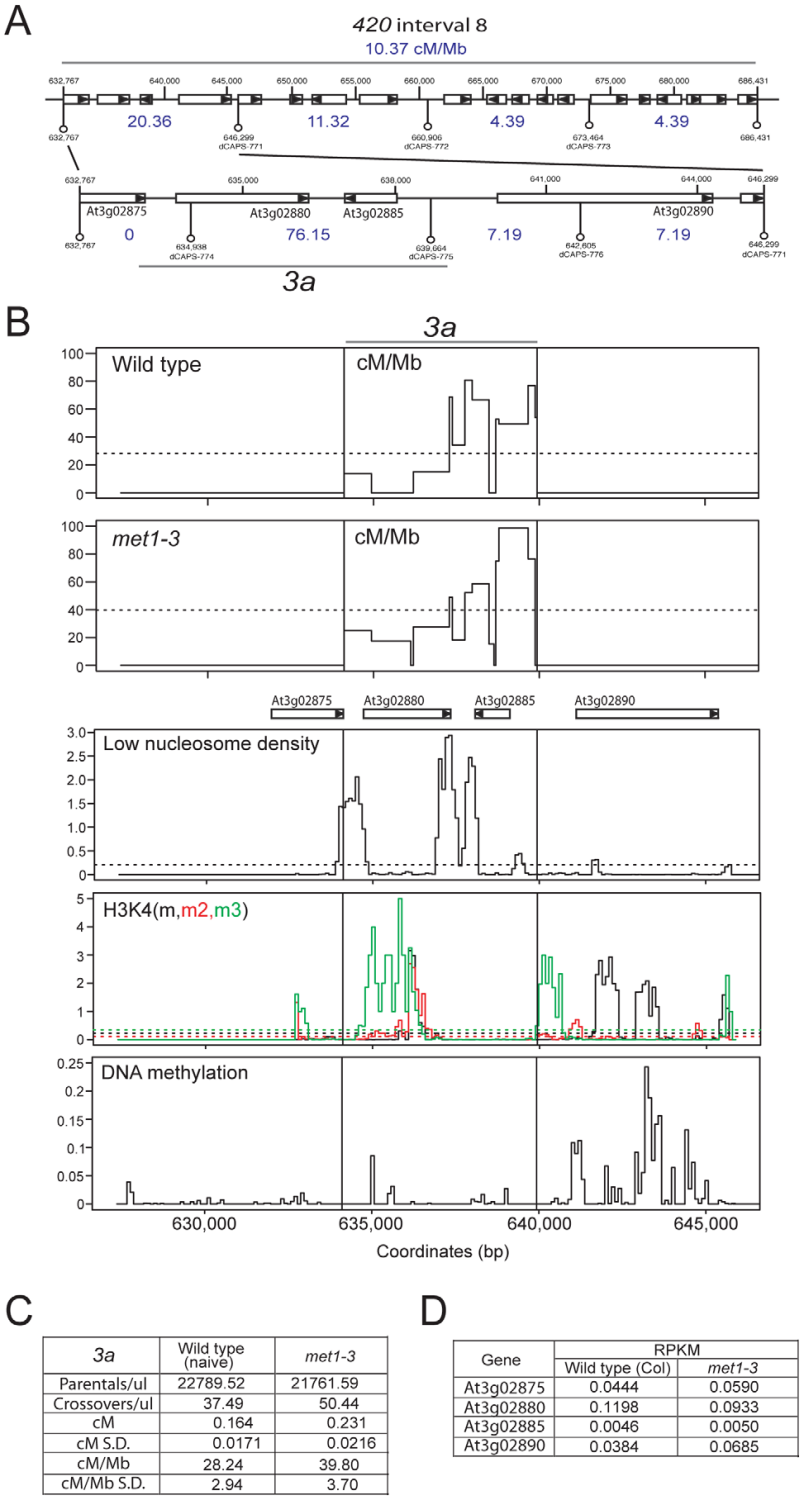
Elevated crossover hotspot *3a1* activity in *met1–3*. (A) CO frequency distributions (cM/Mb, blue) within *420* map interval 8 measured by dCAPs PCR marker segregation (white bars represent genes, with triangles indicating strand). (B) Plots of cM/Mb for the *3a* CO hotspot shown for wild type and *met1–3^−/−^.* Vertical black lines indicate the position of the inner PCR primers used to amplify *3a*. Epigenomic annotation of the *3a* region with plots displaying low nucleosome density, histone H3K4m (black), H3K4m2 (red) H3K4m3 (green) and DNA methylation densities. (C) Table summarizing quantification of *3a* parental and CO molecule amplifications from pollen genomic DNA and calculation of cM, cM/Mb and associated standard deviations (S.D.). (D) RNA-seq RPKM (total counts mapping to gene/length of gene×total mapped reads, multiplied by 10^6^) for *3a* associated genes in wild type (Col) and *met1–3*. See also Table S10.

Mammalian and fungal meiotic recombination hotspots are typically ∼1–2 kb and display higher DSB and CO frequencies than surrounding regions [40], [41], [76], [77]. To identify CO hotspots within *420* we designed dCAPs PCR markers to define CO distributions at finer-scale within an active interval (interval 8, 10.37 cM/Mb) (Figure 7A) [78]. This defined a 6,708 bp sub-interval with a CO frequency of 76.15 cM/Mb (Figure 7A). To identify CO locations within this interval we used a ‘pollen-typing’ strategy, whereby nested allele-specific PCR primers are used to amplify CO molecules from Col/Ler F_1_ pollen genomic DNA (Text S1 and Figure S2) [79], [80]. We amplified and quantified parental versus CO molecules within a subinterval we call *3a* (Figure 7B, 7C and Table S10). In naïve wild type *3a* has a genetic distance of 0.164 cM (S.D. = 0.0171) and a CO rate of 28.24 cM/Mb (S.D. = 2.94) (Figure 7B, 7C and Table S10). We amplified single CO molecules and genotyped for internal Col/Ler polymorphisms to identify CO locations. Within the *3a* amplicon we observe a complex distribution of CO frequency, with three distinct CO hotspots, each separated by at least one marker interval with 0 CO (hotspot #1: 634109–636119 bp; hotspot #2: 636199–638483 bp; hotspot #3: 638687–639664 bp) (Figure 7B and Table S10). The hotspot peaks overlap with low nucleosome density regions located at the 5′- and 3′-ends of a pair of convergently transcribed genes At3g02880 and At3g02885 (Figure 7B) [28]. The central hotspot has a width of 2,284 bp and a peak activity of 80.81 cM/Mb (Figure 7B, 7C and Table S10), which is 17 fold greater than the chromosome 3 male average (4.76 cM/Mb) (Table S1) [21]. We used epigenomic annotation of this region to investigate the presence of chromatin features associated with *3a* (Figure 7B). The genes associated with *3a* are Pol II transcribed and At3g02875, At3g02880 and At3g02890 posses H3K4me, me2 and me3 within their open reading frames (Figure 7B and 7D) [25], [27]. Low levels of DNA methylation are detected within *3a*, though At3g02890 shows gene-body DNA methylation, consistent with active transcription (Figure 7B) [23].

We next tested *met1–3^−/−^* Col/Ler F_1_ pollen genomic DNA and observed a significant increase in *3a* CO frequency to 39.79 cM/Mb (S.D. = 3.70) compared to wild type 28.24 cM/Mb (S.D. = 2.94) (p_t_ = 5.66×10^−5^) (Figure 7B, 7C and Table S10). Although hotspots locations are similar between wild type and *met1–3^−/−^* the relative proportions of COs observed between the three hotspots are significantly different (Figure 7B and Table S10). Hotspot #1 shows significantly more COs (chi-square p = 0.037), hotspot #2 showed significantly less COs (chi-square p = 0.011), whereas hotspot #3 showed no significant difference (chi-square p = 0.560). This demonstrates that although the *3a* region has a significantly elevated overall CO frequency in *met1–3^−/−^*, the individual hotspots within this region respond differently. This may indicate compensatory interactions, related to observations in *S.cerevisae* where changes in local DSB frequency can alter DSB activity in adjacent regions [81], [82], [83], [84], [85], [86]. Importantly, the genes associated with *3a* do not show significant expression changes in *met1–3^−/−^* relative to wild type in floral tissue, indicating that local Pol II accessibility is unlikely to be altered (Figure 7D) [25]. This is consistent with elevated *3a* hotspot activity in *met1–3^−/−^* being mediated via remodeling driven by increased centromere-proximal COs.

## Discussion

CO frequency is highly variable within the genomes of eukaryotes and local rates are determined by hierarchically interacting mechanisms. Here we demonstrate that domains of epigenetic information, specifically heterochromatic DNA methylation, are important for determining chromosomal patterns of CO frequency in *A. thaliana*. Wild type COs are less frequent in densely DNA methylated, transcriptionally silent regions close to the *A. thaliana* centromeres. These regions show dramatic elevations in Pol II transcription in *met1–3^−/−^* [23], [25], [29], [30]. We speculate that SPO11 accessibility similarly increases in *met1–3^−/−^*, leading to elevated DSBs and COs in the centromeric regions. Immunohistochemistry in *A. thaliana* indicates that SPO11 recruitment to the chromosome and the formation of DSBs, as indicated by γH2A.X foci, are temporally distinct [68]. This may reflect activation of the DSB machinery during axis maturation and tethering of chromatin loops [4], [6], [68]. Hence, it will be important to determine the dynamics of axis maturation to fully understand the changes in CO frequency observed in *met1^−/−^*. It is also possible that additional steps in the recombination pathway are sensitive to chromatin state. For example, if interhomolog strand invasion mediated by the recombinase DMC1 were inhibited by DNA methylation, this might lead to increased use of the homologous centromeric region as a repair template in *met1^−/−^*. Additionally, SPO11 is recruited to DNA following pre-meiotic S-phase and heterochromatin replicates later than euchromatin in *A. thaliana* mitotic cells [87], [88], [89]. Therefore, if heterochromatin also replicates earlier in *met1–3^−/−^* meiotic S-phase, SPO11 recruitment close to the centromeres may also advance, and thus altered temporal progression could contribute to CO remodeling. Hence, a complete understanding of the changes in CO frequency in *met1–3^−/−^* will require future study of many aspects of the meiotic recombination mechanism.

COs frequency and distribution are finely controlled. For example, the CO interference pathway inhibits the formation of adjacent CO events in a distance-dependent manner. In *Caenorhabditis elegans* strong interference leads to one CO per bivalent, independent of the physical length of the chromosome [90]. In *A. thaliana* the majority (85–90%) of COs (type-I) are derived from an interference-sensitive pathway, while the remaining events (type-II) are distributed randomly. In *met1–3^−/−^* we observe an increase in centromere-proximal COs, coupled to pericentromeric decreases and distal euchromatic increases, though total CO numbers are similar to wild type. As DNA methylation is most dramatically lost in the centromeric regions, we hypothesize that increases in recombination in these regions drive CO frequency remodeling. Specifically, increases in *met1–3^−/−^* centromeric COs would inhibit adjacent events in the pericentromeric regions via CO interference. In addition to interference, COs are known to be controlled by a homeostatic pathway. For example, reductions of DSB frequency in *S.cerevisiae* do not lead to proportional reductions in CO frequency, indicating compensatory mechanisms that maintain CO numbers close to a mean [16]. We hypothesize that increases in distal CO frequency in *met1–3^−/−^* arise as a consequence of related homeostatic mechanisms maintaining total CO numbers, at the expense of the pericentromeric regions. Extensive data in *S.cerevisiae* demonstrate that DSB frequency can also be influenced by changes in DSB activity in adjacent regions, over distances up to 60 kb [81], [82], [83], [84], [85], [86]. Similar effects could also contribute to the observed changes in *met1–3* ^−/−^ CO frequencies, driven by elevated DSB frequency in hypomethylated regions. Therefore, changes in *met1–3* ^−/−^ recombination frequency could be caused by both additional and redistributed DSBs. Although, DNA methylation, gene density and gene-associated chromatin strongly correlate with CO frequency in the pericentromeres, this is not the case in the chromosome arms. Other levels of meiotic chromosome organization may be dominant in the distal chromosome arms, for example the meiotic axis [4], [6], [7], [91]. However, it is also possible that loss of DNA methylation from gene bodies or local repeats contributes to changes in *met1–3^−/−^* CO frequency in the chromosome arms [23], [25], [29], [30].

Our *420* genetic maps provide evidence of pronounced heterogeneity of CO rate within *A. thaliana* gene-rich euchromatin. We identify a novel CO hotspot *3a* within this region, which overlaps with intergenic regions of low nucleosome density. Although our hotspot comparisons are made with mitotic epigenomic datasets, in yeast and mammals the majority of low nucleosome density regions are similar between meiotic and mitotic cells [92], [93], [94]. The *3a* hotspot shows elevated activity in *met1–3^−/−^*, though without local change in Pol II transcription. Elevated *3a* activity is consistent with CO remodeling driven by increased centromere-proximal COs in *met1–3^−/−^*. The *3a* hotspot shares many similarities with DSB hotspots defined in *S.cerevisiae*, which occur at LNDs with high SPO11 accessibility and active epigenetic modifications including H3K4me3 [37], [40], [41], [95]. However, low nucleosome density regions and H3K4 methylation are shared between *3a* and many genes. Therefore, we predict that these features are necessary but not sufficient for hotspot activity. Specifically, regional factors such as axis structure or proximity to the telomere may predispose locally permissive chromatin to undergo CO. In humans and mice the PRDM9 zinc-finger H3K4 histone methyltransferase positions CO hotspots to specific *cis-*sequences [36], [96], [97], [98], [99], [100], [101]. As PRDM9 has yet to be identified outside of animals, CO hotspots in yeast and plants may represent a more ancestral pattern within eukaryotes [102]. Although the logic of epigenetic control is conserved throughout the eukaryotes, the distributions and uses of specific chromatin marks can vary. As meiosis originated early during eukaryotic evolution it will be interesting to determine similarities in hotspot specification and the relative contributions of epigenetic information to control of CO frequency within distinct lineages. Together our data demonstrates how epigenetic organization contributes to the hierarchy of CO control mechanisms in plant genomes.

Note added in proof: Decreased pericentromeric and elevated euchromatic CO frequencies have been observed in *ddm1* and *met1* mutant backgrounds, consistent with our observations [103], [104].

## Materials and Methods

### Statistical methods

The R Statistical Language was used for analysis and graphs [105]. Correlations were performed using Pearson's product moment correlation. Comparisons between groups were made using t-tests (p_t_) or, in the case of inter-CO distances, the Wilcoxon-rank sum test (p_w_). Comparisons between proportions were made using chi-square tests. Comparisons of variance between groups were made using F-tests. Using glm, a model was fitted to the counts in Figure 2C including the effects of genotype and chromosomes and with the number of plants and chromosome lengths as offsets. Backward elimination was used to arrive at a parsimonious model, which included the effect of genotype and chromosomes 3 and 4. The p-value for genotype from this final model is given in Figure 2C. The R function glm was used to fit a quasi-Poisson model to the data presented in Tables S4 and S5, using genotype as the predictor. The p-value (p_mod_) for genotype is presented in the tables. The fit of MLH1 count data to the Poisson distribution was performed using the R goodfit function within the vcd package.

### Plant materials and growth conditions

All plants were cultivated on commercial soil and grown in controlled environment chambers at 20°C, 60% humidity with a long day photoperiod (16 hours light) with a light intensity of 150 µmols.

### Pollen tetrad and seed fluorescent scoring

Pollen tetrad and seed fluorescence were assayed as described [60], [70]. For a detailed discussion of pollen flow cytometry see Text S1 and Figure S1.

### PCR and bead array genotyping

Genomic DNA was extracted from leaves using the CTAB method and genotyped using either PCR, an Illumina Beadarray or KASPar technology. Pollen genomic DNA was extracted as described [79]. For a detailed discussion of pollen-typing experiments see Text S1, Figure S2 and Table S11.

### Immunocytology

Meiotic cells were analyzed from staged anthers by immunostaining as described [17].

## Supporting Information

Figure S1 Flow cytometry analysis of *I1b QRT1* pollen fluorescence. (A) Schematic diagram showing homologous chromosomes (black lines) heterozygous for *cis-*linked *FTL*-*eYFP* (green triangles) and *FTL-DsRed* (red triangles) transgenes segregating through meiosis-I and –II in the absence (left) or presence (right) of a crossover (CO) between the transgenes. (B) Micrographs of *I1b/−− QRT1* pollen taken under brightfield (BF) or GFP2-filtered UV (R+G) illumination showing segregation of red and green fluorescence. (C) Histogram displaying characteristics of pollen grains analyzed for forward scatter (FSC) and side scatter (SSC). Pollen grains in gate R1 were selected for further analysis. (D) Pollen grains in gate R1 were analyzed for pulse width/pulse area to exclude events that represent more than one cell and gated in R2. (E) Gate R2 pollen grains from non-transgenic Col analyzed for FL1-H (eYFP) and FL2-H (DsRed) fluorescence intensity showing a majority of non-fluorescent pollen. The proportion of pollen grains occupying each gate is indicated by the values associated with grey crosses. (F) Pollen grains from *FTL567* (eYFP) homozygotes with a majority of yellow fluorescent grains. (G) Pollen grains from *FTL1262* (DsRed) homozygotes with a majority of red fluorescent grains. (H) Pollen grains from *FTL567-FTL1262* (eYFP-DsRed) homozygotes with a majority of red and yellow fluorescent grains. (I) Pollen grains from *I1b FTL567-FTL1262* (eYFP-DsRed) *cis*-linked heterozygotes. Non-recombinant pollen grains are non-fluorescent (R5) or red and yellow fluorescent (R4), whereas recombinant pollen grains are red (R3) or yellow (R5) fluorescence.
(TIF)

Figure S2 Pollen-typing analysis of *3a.* (A) Schematic diagram illustrating pollen-typing strategy. Black lines represent the chromosome with Col and Ler polymorphisms indicated by white or black circles respectively. Nested amplifications using allele-specific primers (arrows) are performed to amplify parental or CO molecules as indicated. (B) Ethidium bromide stained agarose gel showing PCR products from amplifications using allele-specific primers (6339CoF, 6339LeF, 6341CoF, 6341LeF) in combination with a non-allele specific universal primer (6431UR). Amplification products are specific to either Col or Ler genomic DNA templates and are shown for a gradient of annealing temperatures. (C) Nested allele-specific PCR amplification products are specifically seen from genomic DNA from Col/Ler F_1_ hybrid pollen and not from leaf. Amplifications were performed from serial dilutions of DNA containing varying amounts of parental molecules. (D) Example of nested allele specific PCR amplifications from diluted Col/Ler F_1_ pollen DNA. The numbers of negative and positive amplifications at specific DNA dilutions for recombinant and crossover molecules are used to estimate cM/Mb. The majority of amplification products at these dilutions correspond to single crossover molecules, which can be identified by sequencing and internal polymorphism genotyping.
(TIF)

Table S1 Physical and genetic dimensions of the *A. thaliana* genome. Gene and repeat annotations were downloaded from the TAIR10 genome release. Genetic map lengths (cM) are from (1) Col×Ler male backcross (Giraut et al., 2011) [21], (2) Col×Ler female backcross (Giraut et al., 2011) [21], (3) sex averaged map (Giraut et al., 2011) [21], and (4) merged genetic map from 17 F_2_ populations (Salome et al, 2011a, 2011b) [22], [55].
(DOCX)

Table S2 Maps of cM/Mb, gene, repeat and DNA methylation, LND and H3K4me3 densities throughout the *A. thaliana* genome. The coordinates correspond to those of the merged genetic map. The CEN column indicates whether an interval is located in the pericentromeres (Y) or chromsome arms (N).
(XLSX)

Table S3 Tetrad scoring data for *CEN3 qrt1.* NPD = non-parental ditype, T = tetratype. Map distance (cM) = (100 (6N+T))/(2(P+N+T)). Standard error of cM (S.E.) = Sqrt(0.25Var[T/Total]+9Var[N/Total]+3Cov[T/Total,N/Total]). Standard deviation of map distances in each genotype group (S.D.).
(DOCX)

Table S4 Total genetic map length in wild type and *met1–3*. The upper sub-table shows crossover numbers (COs) observed in wild type and *met1–3* recombinants per chromosome and total. The lower sub-table shows the number of double CO pairs (DCOs) observed in each population and the average inter-CO distance (bp) for each chromosome and the whole genome.
(DOCX)

Table S5 MLH1 counts in wild type and *met1–3^−/−^*. Summary of MLH1 counts showing number of meiocytes (N) scored for Col and *met1–3^−/−^* genotypes at diplotene and diakinesis meiotic stages. The p-value from the model fitted using the R glm function compares Col and *met1–3^−/−^* at equivalent stages. The goodness-of-fit of the count data with the Poisson distribution was tested using the R function goodfit in package vcd. The index of dispersion is the variance of the counts divided by their means.
(DOCX)

Table S6 Tetrad scoring data for *I1b qrt1.* NPD = non-parental ditype, T = tetratype. Map distance (cM) = (100 (6N+T))/(2(P+N+T)). Standard error of cM (S.E.) = Sqrt(0.25Var[T/Total]+9Var[N/Total]+3Cov[T/Total,N/Total]). Standard deviation of map distances in each genotype group (S.D.).
(DOCX)

Table S7 Seed scoring data for *420* Col/Col homozygotes. (G+R)/Total = Rf. (1-SQRT(1–2*Rf))*100 = cM.
(DOCX)

Table S8 Seed scoring data for *420* Col/Ler heterozygotes. Fisher's exact test p-value given for differences between *wild type male and female (Col/Col), **wild type male and female (Col/Ler) and ***wild type male and *met1* male (Col/Ler).
(DOCX)

Table S9 Gene, transposon, and cM/Mb frequencies within the *420* interval.
(DOCX)

Table S10 Crossover distributions within *3a* identified by pollen-typing. SNP positions highlighted in red are iden tical to polymorphisms used to design *420* interval 8 dCAPs markers 774 and 775.
(DOCX)

Table S11 Oligonucleotides used for dCAPs markers and *3a* pollen typing. Where relevant the Col/Ler polymorphisms are listed, in addition to being highlighted in the allele-specific PCR primers. Red indicates Col-specific polymorphisms and blue indicates Ler-specific polymorphisms. Green indicates mismatches added to both primer variants and underlined cytosines were added to increase GC content and primer specificity.
(DOCX)

Text S1 Supplemental Experimental Procedures. Additional experimental methods for recombination map analysis, flow cytometry of *I1b QRT1 FTL* pollen, and pollen-typing analysis of the *3a* hotspot.
(DOCX)
