# Supplementary figures and images for: Epigenetic Remodeling of Meiotic Crossover Frequency in Arabidopsis thaliana DNA Methyltransferase Mutants
Source: PLoS Genet. 2012 Aug 2;8(8):e1002844. doi: 10.1371/journal.pgen.1002844 (PMC3410864; doi:10.1371/journal.pgen.1002844)

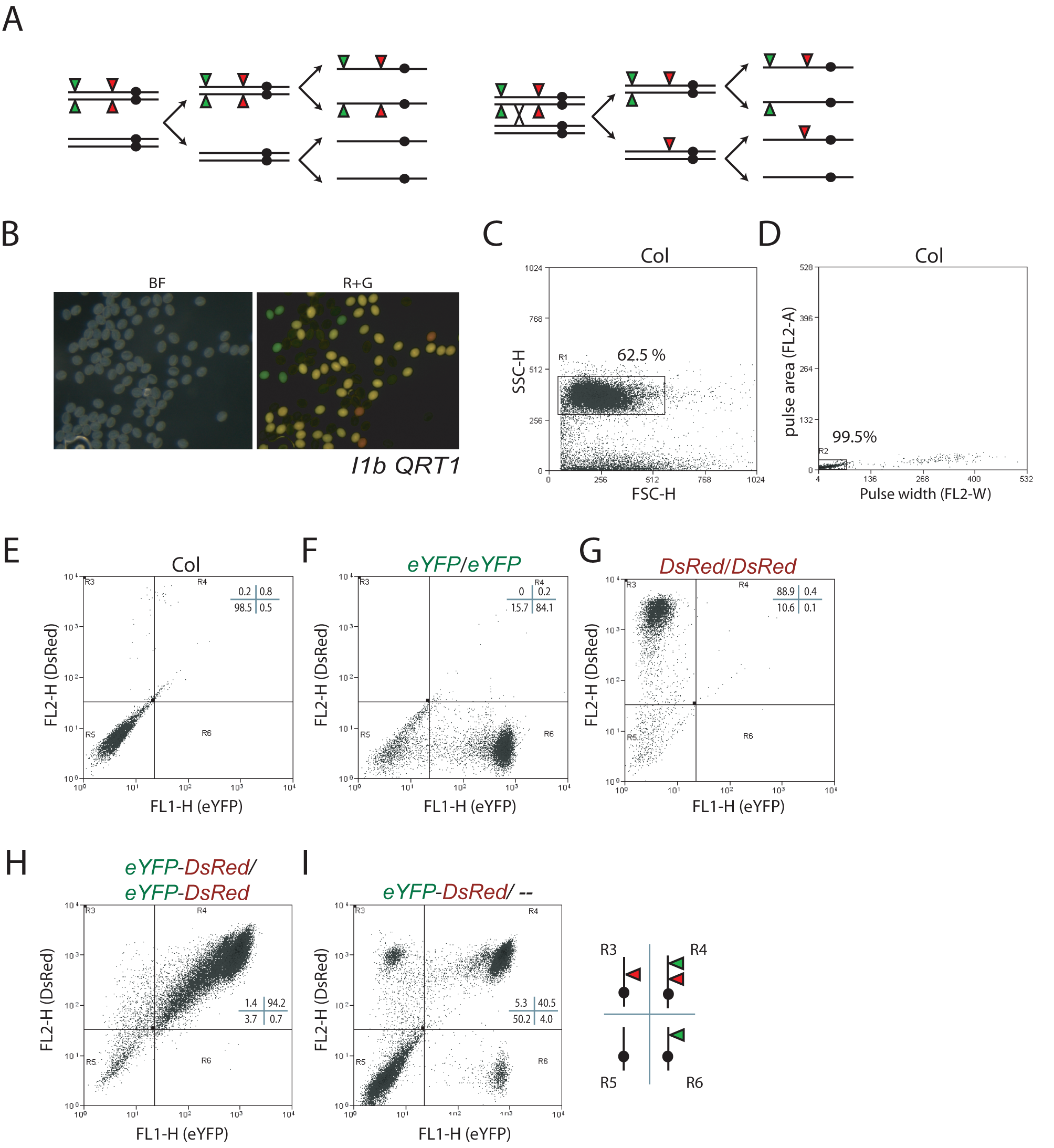

Supplement: Figure S1 — Flow cytometry analysis of I1b QRT1 pollen fluorescence. (A) Schematic diagram showing homologous chromosomes (black lines) heterozygous for cis-linked FTL-eYFP (green triangles) and FTL-DsRed (red triangles) transgenes segregating through meiosis-I and –II in the absence (left) or presence (right) of a crossover (CO) between the transgenes. (B) Micrographs of I1b/−− QRT1 pollen taken under brightfield (BF) or GFP2-filtered UV (R+G) illumination showing segregation of red and green fluorescence. (C) Histogram displaying characteristics of pollen grains analyzed for forward scatter (FSC) and side scatter (SSC). Pollen grains in gate R1 were selected for further analysis. (D) Pollen grains in gate R1 were analyzed for pulse width/pulse area to exclude events that represent more than one cell and gated in R2. (E) Gate R2 pollen grains from non-transgenic Col analyzed for FL1-H (eYFP) and FL2-H (DsRed) fluorescence intensity showing a majority of non-fluorescent pollen. The proportion of pollen grains occupying each gate is indicated by the values associated with grey crosses. (F) Pollen grains from FTL567 (eYFP) homozygotes with a majority of yellow fluorescent grains. (G) Pollen grains from FTL1262 (DsRed) homozygotes with a majority of red fluorescent grains. (H) Pollen grains from FTL567-FTL1262 (eYFP-DsRed) homozygotes with a majority of red and yellow fluorescent grains. (I) Pollen grains from I1b FTL567-FTL1262 (eYFP-DsRed) cis-linked heterozygotes. Non-recombinant pollen grains are non-fluorescent (R5) or red and yellow fluorescent (R4), whereas recombinant pollen grains are red (R3) or yellow (R5) fluorescence. (TIF) [file pgen.1002844.s001.tif]

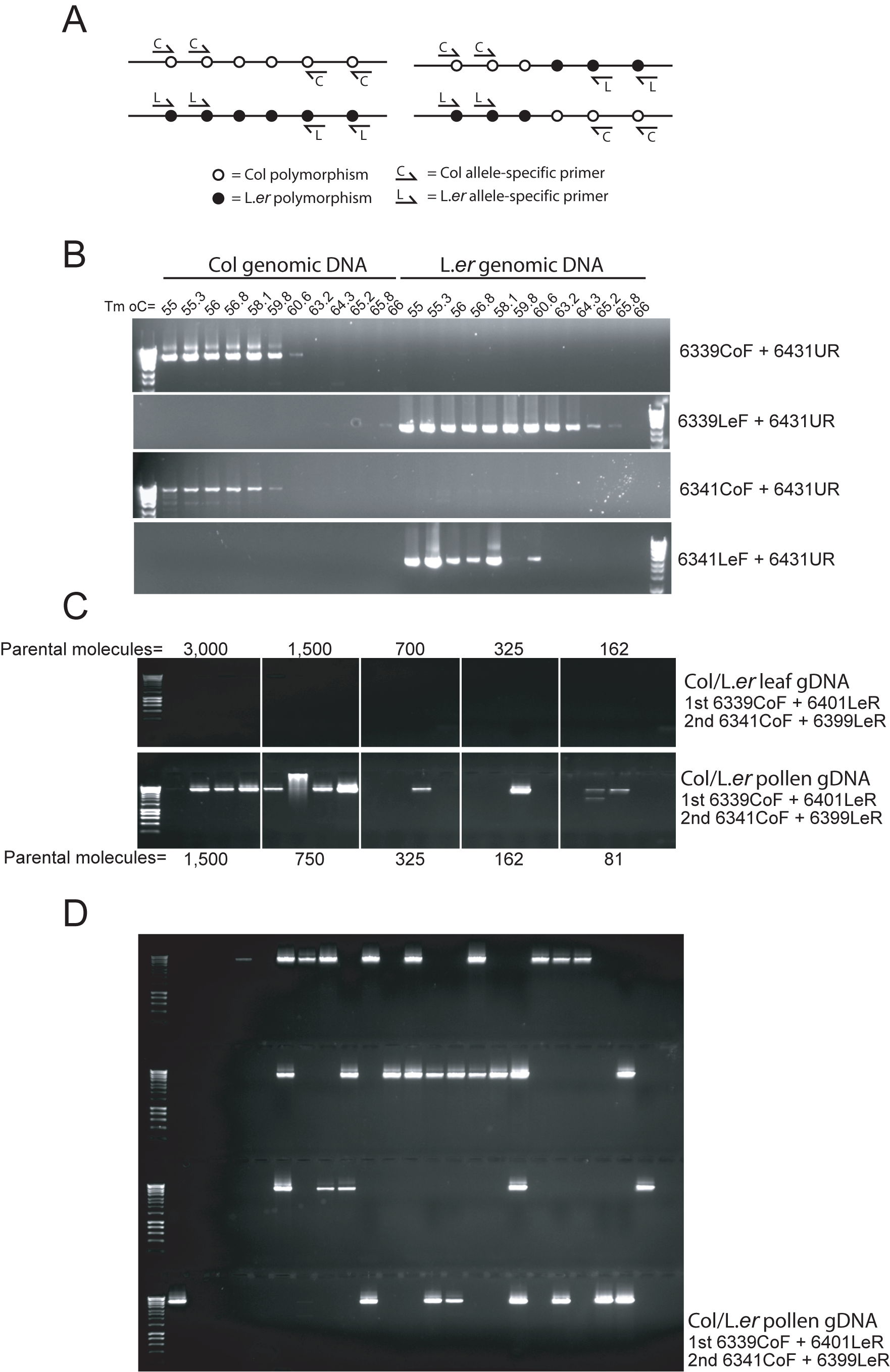

Supplement: Figure S2 — Pollen-typing analysis of 3a. (A) Schematic diagram illustrating pollen-typing strategy. Black lines represent the chromosome with Col and Ler polymorphisms indicated by white or black circles respectively. Nested amplifications using allele-specific primers (arrows) are performed to amplify parental or CO molecules as indicated. (B) Ethidium bromide stained agarose gel showing PCR products from amplifications using allele-specific primers (6339CoF, 6339LeF, 6341CoF, 6341LeF) in combination with a non-allele specific universal primer (6431UR). Amplification products are specific to either Col or Ler genomic DNA templates and are shown for a gradient of annealing temperatures. (C) Nested allele-specific PCR amplification products are specifically seen from genomic DNA from Col/Ler F1 hybrid pollen and not from leaf. Amplifications were performed from serial dilutions of DNA containing varying amounts of parental molecules. (D) Example of nested allele specific PCR amplifications from diluted Col/Ler F1 pollen DNA. The numbers of negative and positive amplifications at specific DNA dilutions for recombinant and crossover molecules are used to estimate cM/Mb. The majority of amplification products at these dilutions correspond to single crossover molecules, which can be identified by sequencing and internal polymorphism genotyping. (TIF) [file pgen.1002844.s002.tif]
