# Supplementary material for: Epigenetic Remodeling of Meiotic Crossover Frequency in Arabidopsis thaliana DNA Methyltransferase Mutants
Source: PLoS Genet. 2012 Aug 2;8(8):e1002844. doi: 10.1371/journal.pgen.1002844 (PMC3410864; doi:10.1371/journal.pgen.1002844)
Supplement: Table S1 — Physical and genetic dimensions of the A. thaliana genome. Gene and repeat annotations were downloaded from the TAIR10 genome release. Genetic map lengths (cM) are from (1) Col×Ler male backcross (Giraut et al., 2011) [21], (2) Col×Ler female backcross (Giraut et al., 2011) [21], (3) sex averaged map (Giraut et al., 2011) [21], and (4) merged genetic map from 17 F2 populations (Salome et al, 2011a, 2011b) [22], [55]. (DOCX) [file pgen.1002844.s003.docx]

**Table S1**

| Chr | Mb | Genes | Repeats | cM (1) | cM (2) | cM(3) | cM(4) |
| --- | --- | --- | --- | --- | --- | --- | --- |
| 1 | 30.43 | 7,509 | 7,135 | 148.2 | 82.6 | 115.4 | 108.9 |
| 2 | 19.70 | 4,470 | 5,949 | 96.4 | 60.4 | 78.4 | 87.5 |
| 3 | 23.46 | 5,650 | 6,461 | 111.8 | 63.3 | 87.5 | 92.9 |
| 4 | 18.59 | 4,308 | 4,967 | 89.3 | 56.2 | 72.8 | 77.3 |
| 5 | 26.98 | 6,559 | 6,677 | 129.1 | 70.4 | 99.8 | 99.1 |
| Total | 119.15 | 28,946 | 31,189 | 574.8 | 332.9 | 453.9 | 465.7 |
|  |  |  |  |  |  |  |  |
| Chr | Mb | Genes/Mb | Repeats/Mb | cM/Mb  (1) | cM/Mb  (2) | cM/Mb  (3) | cM/Mb  (4) |
| 1 | 30.43 | 246.8 | 234.5 | 4.87 | 2.71 | 3.79 | 3.58 |
| 2 | 19.70 | 226.9 | 302.0 | 4.89 | 3.07 | 3.98 | 4.44 |
| 3 | 23.46 | 240.8 | 275.4 | 4.77 | 2.70 | 3.73 | 3.96 |
| 4 | 18.59 | 231.8 | 267.3 | 4.80 | 3.02 | 3.92 | 4.16 |
| 5 | 26.98 | 243.1 | 247.5 | 4.79 | 2.61 | 3.70 | 3.67 |
| Total | 119.15 | 237.8 | 265.3 | 4.82 | 2.79 | 3.81 | 3.91 |
