# Supplementary material for: Epigenetic Remodeling of Meiotic Crossover Frequency in Arabidopsis thaliana DNA Methyltransferase Mutants
Source: PLoS Genet. 2012 Aug 2;8(8):e1002844. doi: 10.1371/journal.pgen.1002844 (PMC3410864; doi:10.1371/journal.pgen.1002844)
Supplement: Table S3 — Tetrad scoring data for CEN3 qrt1. NPD = non-parental ditype, T = tetratype. Map distance (cM) = (100 (6N+T))/(2(P+N+T)). Standard error of cM (S.E.) = Sqrt(0.25Var[T/Total]+9Var[N/Total]+3Cov[T/Total,N/Total]). Standard deviation of map distances in each genotype group (S.D.). (DOCX) [file pgen.1002844.s005.docx]

**Table S3**

| ***CEN3*** | NPD | T | Total | cM | S.E. | S.D. | cM/Mb |
| --- | --- | --- | --- | --- | --- | --- | --- |
| Col | 0 | 126 | 594 | 10.61 | 0.0084 |  | 1.96 |
| Col | 3 | 164 | 793 | 11.48 | 0.0096 |  | 2.13 |
| Col | 2 | 174 | 933 | 9.97 | 0.0077 |  | 1.85 |
| Col | 3 | 205 | 982 | 11.35 | 0.0082 |  | 2.1 |
| Col | 1 | 210 | 916 | 11.79 | 0.0076 |  | 2.18 |
| Col | 1 | 214 | 996 | 11.06 | 0.0071 |  | 2.05 |
| Total | 10 | 1,093 | 5,214 | 11.06 | 0.0033 | 0.66 | 2.05 |
| *MET1* | 0 | 174 | 964 | 9.02 | 0.0062 |  | 1.67 |
| *MET1* | 3 | 211 | 1302 | 8.79 | 0.0064 |  | 1.63 |
| *MET1* | 0 | 324 | 1584 | 10.23 | 0.0051 |  | 1.89 |
| *MET1* | 2 | 189 | 968 | 10.38 | 0.0076 |  | 1.92 |
| *MET1* | 5 | 164 | 918 | 10.57 | 0.0095 |  | 1.96 |
| *MET1* | 2 | 166 | 925 | 9.62 | 0.0077 |  | 1.78 |
| Total | 12 | 1,228 | 6,661 | 9.75 | 0.0028 | 0.74 | 1.81 |
| *met1^+/-^* | 0 | 152 | 1001 | 7.59 | 0.0057 |  | 1.4 |
| *met1^+/-^* | 1 | 172 | 1064 | 8.36 | 0.0063 |  | 1.55 |
| *met1^+/-^* | 0 | 72 | 828 | 4.35 | 0.0049 |  | 0.8 |
| *met1^+/-^* | 1 | 103 | 816 | 6.68 | 0.0068 |  | 1.24 |
| *met1^+/-^* | 0 | 119 | 831 | 7.16 | 0.0061 |  | 1.32 |
| *met1^+/-^* | 1 | 89 | 506 | 9.39 | 0.0102 |  | 1.74 |
| *met1^+/-^* | 0 | 81 | 469 | 8.64 | 0.0087 |  | 1.6 |
| *met1^+/-^* | 5 | 104 | 502 | 13.35 | 0.0157 |  | 2.47 |
| *met1^+/-^* | 1 | 105 | 1000 | 5.55 | 0.0057 |  | 1.03 |
| *met1^+/-^* | 2 | 76 | 532 | 8.27 | 0.0109 |  | 1.53 |
| *met1^+/-^* | 2 | 71 | 535 | 7.76 | 0.0107 |  | 1.44 |
| *met1^+/-^* | 2 | 55 | 503 | 6.66 | 0.0108 |  | 1.23 |
| *met1^+/-^* | 0 | 66 | 508 | 6.5 | 0.0075 |  | 1.2 |
| *met1^+/-^* | 0 | 51 | 508 | 5.02 | 0.0067 |  | 0.93 |
| Total | 15 | 1,316 | 9,603 | 7.32 | 0.0021 | 2.20 | 1.35 |
| *met1^-/-^* | 1 | 89 | 918 | 5.17 | 0.0058 |  | 0.96 |
| *met1^-/-^* | 0 | 114 | 987 | 5.78 | 0.0051 |  | 1.07 |
| *met1^-/-^* | 4 | 145 | 1029 | 8.21 | 0.0079 |  | 1.52 |
| *met1^-/-^* | 4 | 130 | 1050 | 7.33 | 0.0076 |  | 1.36 |
| Total | 9 | 478 | 3,984 | 6.68 | 0.0034 | 1.40 | 1.24 |
| *met1*-self | 0 | 68 | 883 | 3.85 | 0.0045 |  | 0.71 |
| *met1*-self | 1 | 99 | 951 | 5.52 | 0.0058 |  | 1.02 |
| *met1*-self | 1 | 133 | 934 | 7.44 | 0.0065 |  | 1.38 |
| *met1*-self | 2 | 209 | 935 | 11.82 | 0.0081 |  | 2.19 |
| *met1*-self | 2 | 111 | 937 | 6.56 | 0.0069 |  | 1.21 |
| *met1*-self | 0 | 133 | 762 | 8.73 | 0.0069 |  | 1.61 |
| *met1*-self | 2 | 148 | 833 | 9.6 | 0.0083 |  | 1.78 |
| *met1*-self | 0 | 98 | 866 | 5.66 | 0.0054 |  | 1.05 |
| *met1*-self | 4 | 109 | 1215 | 5.47 | 0.0064 |  | 1.01 |
| *met1*-self | 0 | 25 | 1139 | 1.1 | 0.0022 |  | 0.2 |
| Total | 12 | 1,133 | 9,455 | 6.37 | 0.002 | 3.03 | 1.18 |
