# Supplementary material for: Epigenetic Remodeling of Meiotic Crossover Frequency in Arabidopsis thaliana DNA Methyltransferase Mutants
Source: PLoS Genet. 2012 Aug 2;8(8):e1002844. doi: 10.1371/journal.pgen.1002844 (PMC3410864; doi:10.1371/journal.pgen.1002844)
Supplement: Table S4 — Total genetic map length in wild type and met1–3. The upper sub-table shows crossover numbers (COs) observed in wild type and met1–3 recombinants per chromosome and total. The lower sub-table shows the number of double CO pairs (DCOs) observed in each population and the average inter-CO distance (bp) for each chromosome and the whole genome. (DOCX) [file pgen.1002844.s006.docx]

**Table S4**

|  | Wild type (n=95) | | | *met1-3* (n=95) | | |
| --- | --- | --- | --- | --- | --- | --- |
| Chr | COs | mean | S.D. | COs | mean | S.D. |
| 1 | 107 | 1.13 | 0.79 | 88 | 0.93 | 0.93 |
| 2 | 71 | 0.75 | 0.68 | 70 | 0.74 | 0.70 |
| 3 | 96 | 1.01 | 0.75 | 90 | 0.95 | 0.66 |
| 4 | 67 | 0.71 | 0.74 | 61 | 0.64 | 0.80 |
| 5 | 109 | 1.15 | 0.88 | 104 | 1.09 | 0.63 |
| Total | 450 | 4.74 | 1.73 | 413 | 4.35 | 1.76 |
|  | Wild type (n=95) | | | *met1-3* (n=95) | | |
| Chr | DCOs | InterCO distance | | DCOs | InterCO distance | |
| 1 | 57 | 13,040,147 | | 37 | 15,043,019 | |
| 2 | 20 | 8,567,102 | | 17 | 8,626,655 | |
| 3 | 45 | 10,143,844 | | 45 | 11,779,197 | |
| 4 | 25 | 7,839139 | | 16 | 8,689,633 | |
| 5 | 65 | 12,822,903 | | 52 | 11,015,674 | |
| Average | 212 | 11,323,940 | | 167 | 11,630,670 | |
