# Supplementary material for: Epigenetic Remodeling of Meiotic Crossover Frequency in Arabidopsis thaliana DNA Methyltransferase Mutants
Source: PLoS Genet. 2012 Aug 2;8(8):e1002844. doi: 10.1371/journal.pgen.1002844 (PMC3410864; doi:10.1371/journal.pgen.1002844)
Supplement: Table S5 — MLH1 counts in wild type and met1–3−/−. Summary of MLH1 counts showing number of meiocytes (N) scored for Col and met1–3−/− genotypes at diplotene and diakinesis meiotic stages. The p-value from the model fitted using the R glm function compares Col and met1–3−/− at equivalent stages. The goodness-of-fit of the count data with the Poisson distribution was tested using the R function goodfit in package vcd. The index of dispersion is the variance of the counts divided by their means. (DOCX) [file pgen.1002844.s007.docx]

**Table S5**

| Genotype | Stage | N | Mean foci | St.Dev | p-value  (model-fit) | p-value  (Poisson test) | Index of dispersion |
| --- | --- | --- | --- | --- | --- | --- | --- |
| Col | Diplotene | 19 | 7.26 | 2.26 |  | 9.8 e-3 | 0.70 |
| Col | Diakinesis | 37 | 9.3 | 2.31 |  | 8.7 e-3 | 0.57 |
| *met1-3^-/-^* | Diplotene | 61 | 9.33 | 2.26 | 9.1 e-4 | 1.7 e-3 | 0.55 |
| *met1-3^-/-^* | Diakinesis | 11 | 8.64 | 1.96 | 0.39 | 1.9 e-2 | 0.45 |
