# Supplementary material for: Epigenetic Remodeling of Meiotic Crossover Frequency in Arabidopsis thaliana DNA Methyltransferase Mutants
Source: PLoS Genet. 2012 Aug 2;8(8):e1002844. doi: 10.1371/journal.pgen.1002844 (PMC3410864; doi:10.1371/journal.pgen.1002844)
Supplement: Table S6 — Tetrad scoring data for I1b qrt1. NPD = non-parental ditype, T = tetratype. Map distance (cM) = (100 (6N+T))/(2(P+N+T)). Standard error of cM (S.E.) = Sqrt(0.25Var[T/Total]+9Var[N/Total]+3Cov[T/Total,N/Total]). Standard deviation of map distances in each genotype group (S.D.). (DOCX) [file pgen.1002844.s008.docx]

**Table S6**

| ***I1b*** | NPD | T | Total | cM | S.E. | S.D. | cM/Mb |
| --- | --- | --- | --- | --- | --- | --- | --- |
| Col | 0 | 225 | 1411 | 7.97 | 0.0049 |  | 4.31 |
| Col | 0 | 255 | 1512 | 8.43 | 0.0048 |  | 4.56 |
| Col | 3 | 301 | 2033 | 7.85 | 0.0047 |  | 4.24 |
| Col | 2 | 205 | 1269 | 8.55 | 0.0061 |  | 4.62 |
| Total | 5 | 986 | 6,225 | 8.16 | 0.0025 | 0.34 | 4.41 |
| *MET1* | 0 | 246 | 1569 | 7.84 | 0.0046 |  | 4.24 |
| *MET1* | 0 | 180 | 998 | 9.02 | 0.0061 |  | 4.87 |
| *MET1* | 0 | 123 | 982 | 6.26 | 0.0053 |  | 3.39 |
| Total | 0 | 549 | 3,549 | 7.73 | 0.003 | 1.38 | 4.18 |
| *met1^+/-^* | 0 | 226 | 1536 | 7.36 | 0.0045 |  | 3.98 |
| *met1^+/-^* | 0 | 183 | 1057 | 8.66 | 0.0058 |  | 4.68 |
| *met1^+/-^* | 0 | 159 | 930 | 8.55 | 0.0062 |  | 4.62 |
| *met1^+/-^* | 3 | 253 | 1024 | 13.23 | 0.0083 |  | 7.15 |
| *met1^+/-^* | 0 | 130 | 946 | 6.87 | 0.0056 |  | 3.71 |
| *met1^+/-^* | 1 | 174 | 914 | 9.85 | 0.0072 |  | 5.32 |
| *met1^+/-^* | 0 | 187 | 954 | 9.8 | 0.0064 |  | 5.3 |
| Total | 4 | 1,312 | 7,361 | 9.07 | 0.0024 | 2.10 | 4.91 |
| *met1^-/-^* | 1 | 210 | 1000 | 10.8 | 0.0071 |  | 5.84 |
| *met1^-/-^* | 0 | 193 | 1005 | 9.6 | 0.0062 |  | 5.19 |
| *met1^-/-^* | 0 | 188 | 1008 | 9.33 | 0.0061 |  | 5.04 |
| *met1^-/-^* | 0 | 213 | 1006 | 10.59 | 0.0064 |  | 5.72 |
| *met1^-/-^* | 0 | 229 | 976 | 11.73 | 0.0068 |  | 6.34 |
| *met1^-/-^* | 0 | 242 | 1000 | 12.1 | 0.0068 |  | 6.54 |
| *met1^-/-^* | 0 | 237 | 903 | 13.12 | 0.0073 |  | 7.09 |
| Total | 1 | 1,512 | 6,898 | 11.00 | 0.0025 | 1.37 | 5.95 |
| *met1*-self | 1 | 229 | 987 | 11.9 | 0.0073 |  | 6.44 |
| *met1*-self | 0 | 260 | 1027 | 12.66 | 0.0068 |  | 6.84 |
| *met1*-self | 0 | 256 | 1001 | 12.79 | 0.0069 |  | 6.91 |
| *met1*-self | 0 | 185 | 1014 | 9.12 | 0.0061 |  | 4.93 |
| *met1*-self | 2 | 227 | 1000 | 11.95 | 0.0078 |  | 6.46 |
| *met1*-self | 3 | 176 | 894 | 10.85 | 0.0087 |  | 5.86 |
| Total | 6 | 1,333 | 5,923 | 11.56 | 0.003 | 1.37 | 6.25 |
