# Supplementary material for: Epigenetic Remodeling of Meiotic Crossover Frequency in Arabidopsis thaliana DNA Methyltransferase Mutants
Source: PLoS Genet. 2012 Aug 2;8(8):e1002844. doi: 10.1371/journal.pgen.1002844 (PMC3410864; doi:10.1371/journal.pgen.1002844)
Supplement: Table S7 — Seed scoring data for 420 Col/Col homozygotes. (G+R)/Total = Rf. (1-SQRT(1–2*Rf))*100 = cM. (DOCX) [file pgen.1002844.s009.docx]

**Table S7**

| ***420*** | G | R | R+G | -- | Total | cM | S.D. | cM/Mb |
| --- | --- | --- | --- | --- | --- | --- | --- | --- |
| Col | 145 | 173 | 1,250 | 289 | 1,857 | 18.91 |  | 3.70 |
| Col | 189 | 186 | 1,268 | 276 | 1,919 | 21.95 |  | 4.04 |
| Col | 186 | 173 | 1,288 | 306 | 1,953 | 20.48 |  | 3.84 |
| Total | 520 | 532 | 3,806 | 871 | 5,729 | 20.45 | 0.86 | 4.01 |
| *met1-3^+/-^* | 150 | 151 | 872 | 194 | 1,367 | 25.19 |  | 4.93 |
| *met1-3^+/-^* | 194 | 204 | 1,312 | 300 | 2,010 | 22.28 |  | 4.36 |
| *met1-3^+/-^* | 127 | 130 | 803 | 176 | 1,236 | 23.57 |  | 4.62 |
| *met1-3^+/-^* | 204 | 205 | 1,297 | 296 | 2,002 | 23.10 |  | 4.52 |
| *met1-3^+/-^* | 149 | 153 | 977 | 226 | 1,505 | 22.63 |  | 4.43 |
| *met1-3^+/-^* | 156 | 154 | 970 | 223 | 1,503 | 23.35 |  | 4.57 |
| *met1-3^+/-^* | 174 | 171 | 1,116 | 253 | 1,714 | 22.71 |  | 4.45 |
| *met1-3^+/-^* | 172 | 179 | 1,082 | 245 | 1,678 | 23.73 |  | 4.65 |
| Total | 1,326 | 1,347 | 8,429 | 1,913 | 13,015 | 23.24 | 0.90 | 4.55 |
