# Supplementary material for: Epigenetic Remodeling of Meiotic Crossover Frequency in Arabidopsis thaliana DNA Methyltransferase Mutants
Source: PLoS Genet. 2012 Aug 2;8(8):e1002844. doi: 10.1371/journal.pgen.1002844 (PMC3410864; doi:10.1371/journal.pgen.1002844)
Supplement: Table S8 — Seed scoring data for 420 Col/Ler heterozygotes. Fisher's exact test p-value given for differences between *wild type male and female (Col/Col), **wild type male and female (Col/Ler) and ***wild type male and met1 male (Col/Ler). (DOCX) [file pgen.1002844.s010.docx]

**Table S8**

| ***420*** |  |  |  |  |  |  |  |  |  |
| --- | --- | --- | --- | --- | --- | --- | --- | --- | --- |
| Genotype | Gender | Col/Ler | R | G | R+G | -- | Total | cM | p value |
| Wild type | Male | Col/Col | 811 | 800 | 2,454 | 2,485 | 6,550 | 24.59 |  |
| Wild type | Female | Col/Col | 394 | 402 | 2,648 | 2,624 | 6,068 | 13.12 | <2.2x10-^16^ * |
| Wild type | Male | Col/Ler | 767 | 851 | 3,357 | 3,736 | 8,711 | 18.57 |  |
| Wild type | Female | Col/Ler | 236 | 208 | 2,472 | 2,308 | 5,224 | 8.50 | <2.2x10-^16^ ** |
| *met1-3^-/-^* | Male | Col/Ler | 231 | 146 | 545 | 621 | 1,543 | 24.43 | <2.2x10-^16^ *** |
