# Supplementary material for: Epigenetic Remodeling of Meiotic Crossover Frequency in Arabidopsis thaliana DNA Methyltransferase Mutants
Source: PLoS Genet. 2012 Aug 2;8(8):e1002844. doi: 10.1371/journal.pgen.1002844 (PMC3410864; doi:10.1371/journal.pgen.1002844)
Supplement: Table S9 — Gene, transposon, and cM/Mb frequencies within the 420 interval. (DOCX) [file pgen.1002844.s011.docx]

**Table S9**

| Interval | Coordinate | Polymorphism | Genes/Mb | Repeats/Mb | Wild type cM/Mb | *met1-3^-/-^*  cM/Mb |
| --- | --- | --- | --- | --- | --- | --- |
| 1 | 256,516 | NapA::GFP | 411.046 | 0 | 6.177 | 13.625 |
| 2 | 310,038 | A/G | 374.77 | 18.739 | 7.228 | 10.249 |
| 3 | 363,404 | A/G | 318.937 | 18.761 | 5.169 | 5.131 |
| 4 | 416,706 | A/T | 198.976 | 54.266 | 3.987 | 9.893 |
| 5 | 471,989 | A/C | 412.348 | 56.229 | 6.197 | 10.251 |
| 6 | 525,342 | A/T | 355.819 | 74.909 | 1.032 | 5.121 |
| 7 | 578,740 | A/C | 407.204 | 18.509 | 2.04 | 11.811 |
| 8 | 632,767 | A/G | 335.42 | 37.269 | 10.268 | 11.891 |
| 9 | 686,431 | A/G | 296.951 | 55.678 | 6.136 | 8.459 |
| 10 | 740,312 | A/G | 337.939 | 56.323 | 7.242 | 3.423 |
| 11 | 793,576 | G/A | 370.089 | 92.522 | 6.118 | 11.808 |
| 12 | 847,617 | A/G | 243.756 | 37.501 | 12.399 | 5.128 |
| 13 | 900,949 | A/G | 273.378 | 54.676 | 3.013 | 4.984 |
| 14 | 955,818 | A/G | 360.915 | 37.991 | 3.14 | 5.195 |
| 15 | 1,008,462 | A/G | 295.705 | 73.926 | 9.166 | 10.108 |
| 16 | 1,062,570 | A/C | 353.883 | 93.127 | 8.211 | 6.791 |
| 17 | 1,116,260 | A/G | 308.292 | 45.337 | 6.995 | 8.266 |
| 18 | 1,226,545 | A/G | 314.558 | 38.128 | 5.778 | 5.213 |
| 19 | 1,331,454 | A/G | 375.749 | 263.024 | 6.212 | 10.276 |
| 20 | 1,384,681 | A/C | 299.44 | 131.005 | 5.156 | 5.118 |
| 21 | 1,438,114 | A/C | 222.226 | 37.038 | 11.225 | 13.505 |
| 22 | 1,492,113 | A/C | 332.238 | 73.831 | 2.034 | 5.048 |
| 23 | 1,546,291 | A/C | 283.318 | 132.215 | 7.286 | 8.609 |
| 24 | 1,599,235 | C/G | 350.722 | 55.377 | 4.069 | 1.683 |
| 25 | 1,653,409 | A/G | 258.527 | 73.865 | 0 | 0 |
| 26 | 1,707,562 | A/G | 330.148 | 55.025 | 3.032 | 11.704 |
| 27 | 1,762,083 | A/G | 339.226 | 18.846 | 5.192 | 6.872 |
| 28 | 1,815,145 | C/G | 374.932 | 18.747 | 3.099 | 11.962 |
| 29 | 1,868,488 | A/G | 330.56 | 91.822 | 6.072 | 5.022 |
| 30 | 1,922,941 | C/G | 224.585 | 262.015 | 4.125 | 5.118 |
| 31 | 1,976,373 | A/G | 183.884 | 18.388 | 3.04 | 5.029 |
| 32 | 2,030,755 | A/T | 246.801 | 18.985 | 3.138 | 1.731 |
| 33 | 2,083,429 | A/T | 236.437 | 127.312 | 2.004 | 3.316 |
| 34 | 2,138,412 | A/G | 337.819 | 0 | 2.068 | 8.554 |
| 35 | 2,191,695 | A/T | 382.731 | 76.546 | 5.272 | 8.722 |
| 36 | 2,243,951 | C/G | 304.545 | 71.658 | 0 | 0 |
| 37 | 2,299,772 | A/G | 226.492 | 113.246 | 6.24 | 5.162 |
| 38 | 2,352,754 | A/G | 411.592 | 18.709 | 2.062 | 11.938 |
| 39 | 2,406,205 | A/G | 337.743 | 18.763 | 3.102 | 5.131 |
| 40 | 2,459,500 | A/G | 314.343 | 18.491 | 2.038 | 6.742 |
| 41 | 2,513,581 | A/C | 317.064 | 18.651 | 7.194 | 1.7 |
| 42 | 2,567,198 | A/G | 263.41 | 18.815 | 0 | 1.715 |
| 43 | 2,620,347 | A/G | 368.657 | 239.627 | 0 | 6.721 |
| 44 | 2,674,598 | A/G | 299.631 | 0 | 0 | 1.707 |
| 45 | 2,727,997 | A/G | 295.012 | 73.753 | 1.016 | 0 |
| 46 | 2,782,232 | A/G | 289.956 | 57.991 | 3.196 | 0 |
| 47 | 2,833,964 | A/G | 289.677 | 54.314 | 0.998 | 0 |
| 48 | 2,889,198 | A/G | 360.6 | 37.958 | 3.137 | 3.46 |
| 49 | 2,941,888 | A/G | 393.391 | 107.288 | 1.971 | 8.15 |
| 50 | 2,997,812 | A/G | 318.919 | 0 | 4.135 | 1.71 |
| 51 | 3,051,117 | A/G | 353.258 | 92.963 | 8.196 | 11.864 |
| 52 | 3,104,902 | A/G | 281.341 | 187.561 | 3.101 | 0 |
| 53 | 3,158,218 | A/G | 293.567 | 36.696 | 4.044 | 0 |
| 54 | 3,212,720 | A/G | 287.432 | 23.953 | 3.96 | 2.183 |
| 55 | 3,254,469 | A/T | 321.375 | 67.658 | 3.728 | 1.542 |
| 56 | 3,313,590 | C/G | 309.523 | 17.196 | 1.895 | 0 |
| 57 | 3,371,744 | A/G | 417.582 | 18.156 | 5.002 | 1.655 |
| 58 | 3,426,823 | A/T | 348.598 | 128.431 | 3.033 | 6.69 |
| 59 | 3,481,327 | C/G | 281.236 | 74.996 | 4.133 | 6.836 |
| 60 | 3,534,663 | A/G | 354.623 | 149.315 | 1.028 | 3.403 |
| 61 | 3,588,241 | A/T | 333.451 | 74.1 | 1.021 | 5.066 |
| 62 | 3,642,222 | A/C | 244.416 | 112.807 | 10.36 | 6.855 |
| 63 | 3,695,410 | A/G | 378.867 | 227.32 | 2.088 | 5.18 |
| 64 | 3,748,199 | A/G | 200.011 | 109.097 | 2.004 | 0 |
| 65 | 3,803,196 | C/G | 259.991 | 129.996 | 2.047 | 1.693 |
| 66 | 3,857,044 | A/G | 348.247 | 91.644 | 0 | 1.671 |
| 67 | 3,911,603 | A/C | 441.739 | 19.206 | 1.058 | 3.502 |
| 68 | 3,963,670 | A/G | 340.826 | 107.629 | 2.965 | 3.27 |
| 69 | 4,019,417 | C/G | 304.942 | 0 | 2.1 | 0 |
| 70 | 4,071,886 | A/C | 240.812 | 166.716 | 4.083 | 0 |
| 71 | 4,125,870 | A/T | 299.906 | 18.744 | 1.033 | 0 |
| 72 | 4,179,220 | A/G | 280.123 | 112.049 | 0 | 6.809 |
| 73 | 4,232,768 | A/C | 302.675 | 117.364 | 4.085 | 2.252 |
| 74 | 4,394,658 | A/G | 259.129 | 19.933 | 1.098 | 1.817 |
| 75 | 4,444,826 | A/G | 364.109 | 138.708 | 1.911 | 4.742 |
| 76 | 4,502,501 | A/C | 259.37 | 92.632 | 7.146 | 3.378 |
| 77 | 4,556,478 | A/G | 312.322 | 128.603 | 2.025 | 3.349 |
| 78 | 4,610,909 | A/G | 244.669 | 18.821 | 0 | 1.716 |
| 79 | 4,664,042 | A/G | 307.959 | 38.495 | 1.061 | 0 |
| 80 | 4,715,997 | A/G | 201.395 | 91.543 | 3.027 | 6.676 |
| 81 | 4,770,616 | A/G | 329.64 | 91.567 | 6.055 | 8.347 |
| 82 | 4,825,221 | A/G | 190.865 | 95.433 | 1.052 | 0 |
| 83 | 4,877,614 | A/G | 291.407 | 109.278 | 3.011 | 0 |
| 84 | 4,932,520 | A/C | 305.53 | 76.383 | 2.104 | 1.741 |
| 85 | 4,984,888 | T/A | 259.283 | 18.52 | 0 | 0 |
| 86 | 5,038,883 | A/T | 364.884 | 24.326 | 0 | 4.435 |
| 87 | 5,079,992 | G/A | 321.182 | 32.118 | 1.77 | 1.464 |
| 88 | 5,142,262 | A/C | 328.289 | 49.243 | 0.904 | 1.496 |
| 89 | 5,203,184 | A/C | 270.704 | 135.352 | 0 | 3.525 |
| 90 | 5,254,901 | A/C | 323.329 | 38.039 | 0 | 1.734 |
| 91 | 5,307,479 | C/A | 387.754 | 36.929 | 4.07 | 8.416 |
| na | 5,361,637 | NapA::RFP | na | na | na | na |
