# Supplementary material for: Epigenetic Remodeling of Meiotic Crossover Frequency in Arabidopsis thaliana DNA Methyltransferase Mutants
Source: PLoS Genet. 2012 Aug 2;8(8):e1002844. doi: 10.1371/journal.pgen.1002844 (PMC3410864; doi:10.1371/journal.pgen.1002844)
Supplement: Table S10 — Crossover distributions within 3a identified by pollen-typing. SNP positions highlighted in red are iden tical to polymorphisms used to design 420 interval 8 dCAPs markers 774 and 775. (DOCX) [file pgen.1002844.s012.docx]

**Table S10**

| ***3a*** |  | SNP | |  | Wild type | | *met1-3^-/-^* | |
| --- | --- | --- | --- | --- | --- | --- | --- | --- |
|  | Position | Col | L.*er* | length (bp) | Crossovers | cM/Mb | Crossovers | cM/Mb |
| Chr3 | 634109 | A | G | 829 | 7 | 13.65 | 9 | 24.72 |
| Chr3 | 634938 | C | T | 1181 | 0 | 0 | 9 | 17.35 |
| Chr3 | 636119 | A | C | 80 | 0 | 0 | 0 | 0 |
| Chr3 | 636199 | T | A | 1084 | 10 | 14.91 | 13 | 27.31 |
| Chr3 | 637283 | T | A | 94 | 4 | 68.77 | 2 | 48.45 |
| Chr3 | 637377 | - | A | 377 | 8 | 34.30 | 3 | 18.12 |
| Chr3 | 637754 | A | G | 220 | 11 | 80.81 | 5 | 51.76 |
| Chr3 | 637974 | A | G | 509 | 21 | 66.68 | 13 | 15.18 |
| Chr3 | 638483 | A | T | 150 | 0 | 0 | 1 | 0 |
| Chr3 | 638633 | T | A | 6 | 0 | 0 | 0 | 0 |
| Chr3 | 638639 | C | T | 32 | 0 | 0 | 0 | 0 |
| Chr3 | 638671 | C | - | 6 | 0 | 0 | 0 | 0 |
| Chr3 | 638677 | C | T | 2 | 0 | 0 | 0 | 0 |
| Chr3 | 638679 | A | G | 2 | 0 | 0 | 0 | 0 |
| Chr3 | 638681 | A | - | 6 | 0 | 0 | 0 | 0 |
| Chr3 | 638687 | - | T | 92 | 3 | 52.70 | 3 | 74.26 |
| Chr3 | 638779 | - | AT | 885 | 27 | 49.31 | 38 | 97.78 |
| Chr3 | 639664 | C | A | 210 | 10 | 76.96 | 7 | 75.91 |
| Chr3 | 639874 | A | G | 60 | 2 | 53.87 | 0 | 0 |
| Chr3 | 639934 | A | T |  |  |  |  |  |
|  |  |  | Total | 5825 | 99 | 28.24 | 101 | 39.79 |
