# Supplementary material for: Epigenetic Remodeling of Meiotic Crossover Frequency in Arabidopsis thaliana DNA Methyltransferase Mutants
Source: PLoS Genet. 2012 Aug 2;8(8):e1002844. doi: 10.1371/journal.pgen.1002844 (PMC3410864; doi:10.1371/journal.pgen.1002844)
Supplement: Table S11 — Oligonucleotides used for dCAPs markers and 3a pollen typing. Where relevant the Col/Ler polymorphisms are listed, in addition to being highlighted in the allele-specific PCR primers. Red indicates Col-specific polymorphisms and blue indicates Ler-specific polymorphisms. Green indicates mismatches added to both primer variants and underlined cytosines were added to increase GC content and primer specificity. (DOCX) [file pgen.1002844.s013.docx]

**Table S11**

| Name | Sequence (5’-3’) | coordinate | Col | Ler | Enzyme |
| --- | --- | --- | --- | --- | --- |
| 771-F | GAACCAAAGCAGCTTCTTGATTCACACCAT | 646,299 | A | T | *Nde*I |
| 771-R | TGAACAGTCAATGTAGAGCTCTTGCG | 646,299 | A | T | *Nde*I |
| 772-F | GTTTTGAATGAAGTGAATTCAATCTAAACTAG | 660,906 | T | G | *Spe*I |
| 772-R | CACACACATTAGAAAAAAGTAGATGATCTTG | 660,906 | T | G | *Spe*I |
| 773-F | AAAGCTATCTCCAGAGTTTAATATAAGTGTCGA | 673,464 | C | G | *Sal*I |
| 773-R | CTTCGCAAACCTTACAGAGATTTATATTCG | 673,464 | C | G | *Sal*I |
| 774-F | GCGTAAGCTAAGTCTCTCTGTTGTC | 634,938 | C | T | *Nco*I |
| 774-R | GTTCCATAGCAAAGGACGGCCATG | 634,938 | C | T | *Nco*I |
| 775-F | AATTATTAATGGACATCACCATAACAT | 639,664 | C | A | *Nde*I |
| 775-R | ATTTCGTCATCCTAAGATTATTTTCGG | 639,664 | C | A | *Nde*I |
| 776-F | CGTTTTTGAAGTCCAATTCGTTTAATTCCT | 642,605 | T | C | *Xho*I |
| 776-R | TTTGAAAATCCTTCCTTTACTTCAAGG | 642,605 | T | C | *Xho*I |
| 6339CoF | GAGAAACCAACCACTTCT | 633,970-633,987 | CT | TC | na |
| 6339LeF | GAGAAACCAACCACTTTC | 633,971-633,988 | CT | TC | na |
| 6341-0CoF | CCCCCTTTCAAATTGATACAACAA | 634,089-634,109 | A | G | na |
| 6341-0LeF | CCCCCTTTCAAATTGATACAACAG | 634,089-634,109 | A | G | na |
| 6399CoR | CCCAAGTTTTCTTCTCAAGCCT | 639,934-639,952 | T | A | na |
| 6399LeR | CCCAAGTTTTCTTCTCAAGCCA | 639,934-639,952 | T | A | na |
| 6401CoR | GGGGATTCTCTATATCTAGGA | 640,174-640,192 | GGA | C | na |
| 6401LeR | CAAGGATTCTCTATATCTAC | 640,174-640,192 | GGA | C | na |
| 6337UF | GACGCTAGGCGCTGGTAAG | 633,706-633,702 | na | na | na |
| 6431UR | CTCGACCGGGGTACACCATC | 643,164-643,183 | na | na | na |
| 6376UF | GTTCAAGCTTAAAGGGAAATCG | 637,619-637,640 | na | na | na |
| 6377UR | GCCCATGACTCGGTGTAAAT | 637,714-637,733 | na | na | na |
| Seq13R | AATGACAATAAATCACTGCAG | 637,672-637,692 | na | na | na |
| Seq12F | GACAGAGCTGAAACTCTTG | 637,414-637,432 | na | na | na |
| Seq11F | TGAGCATGGGTTAGTGGT | 636,145-636,162 | na | na | na |
| Seq2F | TTGAGATGAGACCTATCAG | 634,923-634,941 | na | na | na |
| Seq3F | TCGCCAACTTGTAGAAACAG | 633,823-633,842 | na | na | na |
| Seq4F | 5’-GCATGTGATCTTTGTGGGC | 632,523-632,541 | na | na | na |
