# Supplementary material for: Epigenetic Remodeling of Meiotic Crossover Frequency in Arabidopsis thaliana DNA Methyltransferase Mutants
Source: PLoS Genet. 2012 Aug 2;8(8):e1002844. doi: 10.1371/journal.pgen.1002844 (PMC3410864; doi:10.1371/journal.pgen.1002844)
Supplement: Text S1 — Supplemental Experimental Procedures. Additional experimental methods for recombination map analysis, flow cytometry of I1b QRT1 FTL pollen, and pollen-typing analysis of the 3a hotspot. (DOCX) [file pgen.1002844.s014.docx]

**Text S1 - Supplemental Experimental Procedures**

**Recombination map analysis**

Genotype data from 17 F_2_ populations was used to construct genetic maps (Kosambi) using R/qtl, which were then merged using MergeMap (Broman et al., 2003; Salome et al., 2011; Wu et al., 2008). The average population size (411) means a genetic distance of >0.5 cM is required for the confidence interval to be less than 1 cM. Therefore the first marker in pairs closer than 80 kb were dropped using R/qtl. MergeMap produced a branching graph in three cases, indicating ambiguity in marker order compared to the Col reference sequence (TAIR10), which may be due to structural differences between accessions (Salome et al., 2011). In these cases the first marker of the ambiguous pairs were dropped in R/qtl. Genetic distances were converted to cM/Mb using physical distances estimated from the Col reference sequence. Correlations were performed using Pearson’s product moment correlation.

**Flow cytometry of *I1b QRT1 FTL* pollen**

*FTL* lines expressing eYFP and DsRed were backcrossed to Col or *met1-3/+* to complement *qrt1* (Figure S1A and S1B). Pollen was collected using a protocol modified after Becker et al., 2003 (Becker et al., 2003). 10-40 inflorescences per genotype were agitated for 10-15 minutes in 30 ml of pollen sorting buffer (PSB: 10 mM CaCl_2_, 1 mM KCl, 2 mM MES, 5% sucrose, pH 6.5 with NaOH) supplemented with Triton X-100 to a final concentration of 0.01% (PSBT). Following agitation, pollen-containing PSBT was filtered through a 40 nm nylon cell strainer (BD Biosciences) and centrifuged at 450 g for 2 min. The supernatant was removed and the pellet containing pollen was washed once with 40 ml of PSB and centrifuged at 450 g for 2 min. The pollen-enriched pellet was resuspended in 200 ml of PSB and analysed on a FACScan flow cytometer (Becton Dickinson), using a 488-nm argon laser for excitation. Fluorescence of eYFP (514/527 nm excitation/emission) and DsRed (563/582 nm excitation/emission) were detected through 525/30 nm (FL1) and 585/42 nm (FL2) band pass filters, respectively. Wild-type Col-0 and transgenic lines homozygous for eYFP (*FTL424*) and/or DsRed (*FTL1262*) transgenes were used to set the color compensation. Data was analysed with Summit V4.3.02 software (Beckman Coulter Inc).

Gating was set as follows; forward scatter (FSC)/ side scatter (SSC) measurements were used to selects events based on their forward angle light scatter (usually volume or size in solid objects) and ninety degree angled light scatter (usually granularity and surface deformity) (Figure S1C). Pulse width/pulse area measurement was used to exclude events that represent more than one pollen grain (Figure S1D). Pollen was then measured for eYFP (FL1-H) and DsRed (FL2-H) fluorescence intensity (Figure S1E-I). In transgenic lines homozygous for eYFP and/or DsRed transgenes we observed more non-fluorescent pollen grains than expected (gate R5) (Figure S1F-H), which we believe represents pollen grains that have lost expression of *FTL* transgenes. In *I1b/--* heterozygotes we would predict that the number of pollen expressing eYFP alone should equal those expressing DsRed alone, as they represent reciprocal crossover products. Likewise, the number of pollen grains expressing both eYFP and DsRed should equal that of non-fluorescent pollen. As we observe more non-fluorescent pollen grains than expected we assume this reflects loss of *FTL* transgene expression and we therefore exclude these events from our analysis (Figure S1I). First we estimate their number (N_NF_-N_RY_) followed by subtraction from total events to give an adjusted total: N_total_-(N_NF_-N_RY_) (NF=non-fluorescent; R=DsRed; Y=eYFP; RY=DsRed and eYFP). To calculate recombination rate we then divide the recombinant class by the adjusted total. Pollen homozygous for both DsRed and eYFP transgenes show a majority of events in gate R4 (red and green), but greater than expected numbers of non-fluorescent or red-alone pollen (Figure S1H). We explain this again by pollen grains that have lost *FTL* transgene expression. For this reason we calculate genetic distance in *I1b/--* heterozygotes using only numbers of eYFP-expressing pollen using; cM=2*((100*N_Y_)/(N_total_-(N_NF_-N_RY_)).

**Pollen-typing analysis of the *3a* hotspot**

Crossover analysis by pollen-typing uses nested allele-specific PCR from Col/Ler F_1_ pollen genomic DNA (Drouaud and Mezard, 2011; Kauppi et al., 2009) (Figure S2A). Col/Ler hybrid plants were grown alongside one another in controlled environment chambers and genomic DNA from pollen and leaf extracted as described (Drouaud and Mezard, 2011). Col/Ler polymorphisms were identified from publically available datasets (http://1001genomes.org/) in the vicinity of the *3a* interval identified from *420* recombinant mapping. Primers were designed to anneal at their 3’-ends to either Col or Ler polymorphisms (Figure S2A and Table S11). Internal mismatches or additional 5’-C residues were added to improve allele-specific amplification (Drouaud and Mezard, 2011; Kauppi et al., 2009). PCR amplifications were performed in 10 μl using 0.025 U ExTaq (Takara, RR011C), 1x ExTaq reaction buffer, 0.5 μM of forward and reverse primers, 250 μM dNTP (Takara, RR011C) and varying quantities of template genomic DNA. Gradient PCR {(94^o^C 2 minutes); (94^o^C 30 seconds, 55-66^o^C 30 seconds, 68^o^C 5 minutes) 30 cycles; (68^o^C 10 minutes)} was performed from Col or Ler homozygous genomic DNA to determine optimal annealing temperature for allele-specific PCR. Specificity and efficiency of amplification were tested using each allele-specific primer in combination with a universal primer from Col and Ler templates (6337UF or 6431UR) (Figure S2B and Table S11) (Drouaud and Mezard, 2011; Kauppi et al., 2009).

To estimate the concentration of amplifiable crossover and parental molecules from genomic DNA two rounds of allele-specific PCR were performed using the following nested primer sets to amplify a ~6.2 kb product.

Crossovers (Col to Ler) : 1^st^ PCR (6339CoF + 6401LeR), 2^nd^ PCR (6341-0CoF + 6399LeR)

Parentals (Col to Col) : 1^st^ PCR (6339CoF + 6401CoR), 2^nd^ PCR (6341-0CoF + 6399CoR)

Specificity of crossover amplification can be tested by performing allele-specific PCR from Col/Ler F_1_ leaf genomic DNA, which lacks crossover products (Figure S2C). Amplification was performed from serial dilutions of genomic DNA until approximately half of PCR reactions were negative (Figure S2D). Conditions for the first PCR reaction were {(94^o^C 2 minutes); (94^o^C 30 seconds, 60^o^C 45 seconds with 0.5^o^C decrease per cycle, 68^o^C 5 minutes) 18 cycles; (68^o^C 10 minutes)}. 10 μl aliquots from the first reaction were diluted 20-fold with 5 mM Tris (pH 7.5) and 1 μl of the dilution used as a template for the second amplification using nested allele-specific primers {(94^o^C 2 minutes); (94^o^C 30 seconds, 59^o^C 45 seconds with 0.5^o^C decrease per cycle, 68^o^C 5 minutes) 8 cycles; (94^o^C 20 seconds, 55^o^C 30 seconds, 68^o^C 5 minutes) 22 cycles; (68^o^C 10 minutes)}.

The frequency of crossover or parental molecules (m) in input DNA is estimated by measuring the proportion of negative amplifications following DNA dilution and Poisson approximation (m = -ln(number of negative reactions/total number of reactions) (Drouaud and Mezard, 2011; Kauppi et al., 2009). Standard deviation was calculated by comparing molecule number estimations at different dilutions (Drouaud and Mezard, 2011; Kauppi et al., 2009). Amplified single crossover molecules were sequenced using internal primers to identify crossover locations by SNP genotypes (Table S11).

**Supplemental reference**

Becker, J.D., Boavida, L.C., Carneiro, J., Haury, M., and Feijo, J.A. (2003). Transcriptional profiling of Arabidopsis tissues reveals the unique characteristics of the pollen transcriptome. Plant Physiol *133*, 713-725.
